# Detecting Borrelia Spirochetes: A Case Study With Validation Among Autopsy Specimens

**DOI:** 10.3389/fneur.2021.628045

**Published:** 2021-05-10

**Authors:** Shiva Kumar Goud Gadila, Gorazd Rosoklija, Andrew J. Dwork, Brian A. Fallon, Monica E. Embers

**Affiliations:** ^1^Division of Immunology, Tulane National Primate Research Center, Tulane University Health Sciences, Covington, LA, United States; ^2^Department of Psychiatry, Columbia University, New York, NY, United States; ^3^Division of Molecular Imaging and Neuropathology, New York State Psychiatric Institute, New York, NY, United States; ^4^Macedonian Academy of Sciences and Arts, Skopje, Macedonia; ^5^Department of Pathology and Cell Biology, Columbia University, New York, NY, United States

**Keywords:** Lyme, Borrelia, dementia, immunofluorescent, PCR, RNA *in situ* hybridization, Lewy body

## Abstract

The complex etiology of neurodegenerative disease has prompted studies on multiple mechanisms including genetic predisposition, brain biochemistry, immunological responses, and microbial insult. In particular, Lyme disease is often associated with neurocognitive impairment with variable manifestations between patients. We sought to develop methods to reliably detect *Borrelia burgdorferi*, the spirochete bacteria responsible for Lyme disease, in autopsy specimens of patients with a history of neurocognitive disease. In this report, we describe the use of multiple molecular detection techniques for this pathogen and its application to a case study of a Lyme disease patient. The patient had a history of Lyme disease, was treated with antibiotics, and years later developed chronic symptoms including dementia. The patient's pathology and clinical case description was consistent with Lewy body dementia. *B. burgdorferi* was identified by PCR in several CNS tissues and by immunofluorescent staining in the spinal cord. These studies offer proof of the principle that persistent infection with the Lyme disease spirochete may have lingering consequences on the CNS.

## Introduction

Neuroborreliosis can occur in up to 15% of patients with Lyme disease, affecting both the central nervous system (CNS) and peripheral nervous system (PNS). The disease of the nervous system can become chronic and debilitating. Prior studies of persistent post-treatment Lyme encephalopathy demonstrated both immune activation in CSF and serum and metabolic and blood flow deficits in the CNS (1–3). While the persistence of the pathogen after antibiotic treatment in humans remains controversial, animal studies have clearly demonstrated its occurrence (4–8). Evidence from experiments performed in mice, dogs and primates have shown that intact spirochetes can persist in the mammalian host after the administration of antimicrobial drugs, and that they can be metabolically viable (9). Studies *in vitro* have demonstrated that persister Borrelia develop stochastically in the presence of microbiostatic antibiotics and that tolerance is enabled by slowed growth (10, 11).

We have recently demonstrated both inflammation and persistence of Borrelia in the CNS and PNS of doxycycline-treated rhesus macaques that were infected with the Lyme disease pathogen (9, 12). In humans, persistence has been studied early after treatment and in Post-Treatment Lyme Disease (PTLD) patients. In one study, skin biopsies were taken from the erythema migrans (EM) lesion and after treatment (~2 mo later). Approximately 1.7% of these were culture-positive and confirmed as the same strain (13, 14). Human xenodiagnoses were also performed in a more recent study. Larval ticks were placed on patients who had EM (early stage) or PTLDS (15). Tick samples were evaluated by PCR and culture; of the 23 patients on whom ticks fed and were recovered, 19 were negative, 2 were indeterminate, and 2 were positive by PCR (1 patient with EM and 1 with PTLDS). Two other studies have indicated that the spirochetes could be cultured from late stage Lyme patients, yet the cultures took many weeks and rounds of subculturing without active growth (16, 17). Thus, in the absence of a reliable detection system, persistent infection in humans remains difficult to assess. One means to address this issue is to interrogate patient tissue for persistent pathogen through the analysis of post-mortem specimens.

In this report, we describe the use of multiple overlapping techniques, including immunofluorescence assay (IFA), RNA *in situ* hybridization (RNAscope), and PCR for detection of Borrelia spirochetes in post-mortem tissues. As example, we describe the detection of *B. burgdorferi* in the brain tissue of a post-mortem donor from the brain repository of the Lyme and Tick-Borne Diseases Research Center at the Columbia University Irving Medical Center. This individual had a history of Lyme disease that appeared to have been successfully treated with antibiotics; 4 years later developed a neurodegenerative disorder leading to dementia.

## Materials and Methods

### Preparation of Tissues

#### NHP Controls

**Figure 1:**
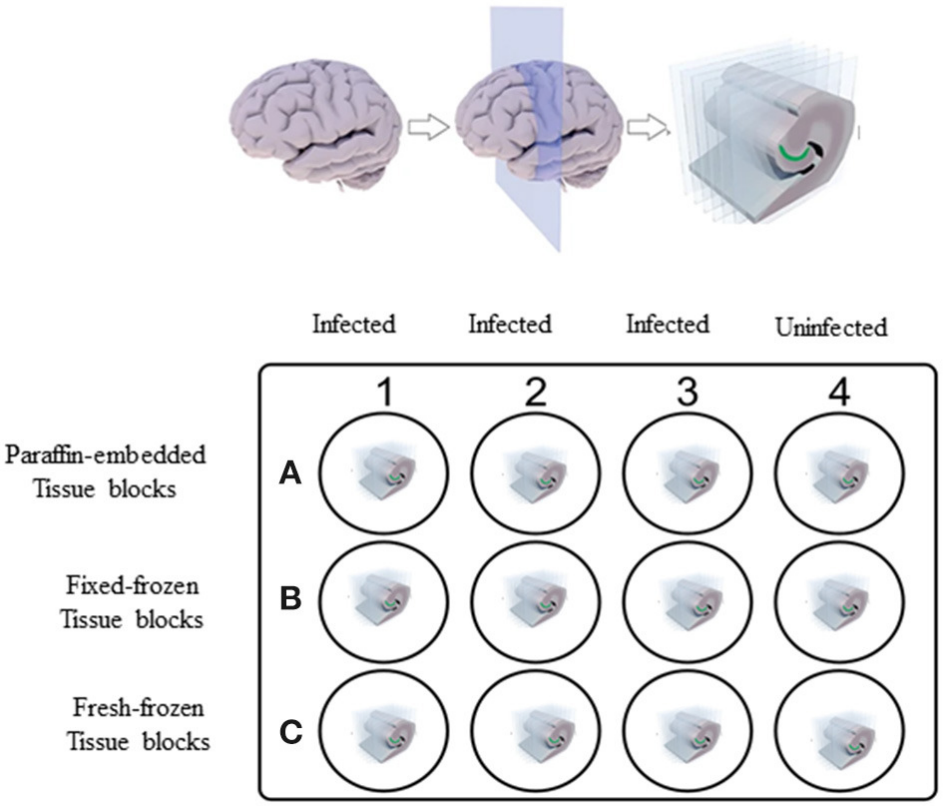
Non-human primate (NHP) control tissue block preparation design. 0.6 cm thick sections of NHP control brain tissues were placed in individual wells of 12-well plate containing 2 ml of RPMI 1640 medium. Tissues in wells A1–A3, B1–B3, and C1–C3 were incubated with 1.0 × 10^7^/ml of spirochetes per well. Tissues in wells A4, B4, and C4 received culture medium alone. After overnight incubation, tissues from wells C1–C4 were washed in PBS and excess buffer was removed using kim wipes and tissues were embedded in OCT and flash-frozen in dry-ice ethanol bath and stored at −80°C. Rest of the tissues in wells A1–A4 and B1–B4 were fixed. Tissues from wells A1–A4 were processed for paraffin-embedded blocks and tissues from wells B1–B4 were processed for Fixed-frozen blocks.

*Ex vivo* experiments were carried out initially on fresh brain tissues collected from non-human primates (NHPs) of Indian origin immediately after euthanasia. These tissues were collected in phosphate-buffered saline (PBS) of pH 7.2 at room temperature. These tissues were then sliced into 0.6 cm sections using a brain tissue slicer and tissue slicing blades and placed in individual wells of a 12-well plate (Figure 1), containing 2 ml of RPMI 1640 medium (18). Nine wells (A1–A3, B1–B3, and C1–C3) out of 12 wells containing the NHP tissue were incubated with *Borrelia burgdorferi* strain B31 clone 5A19 spirochetes (19) at a concentration of 1.0 × 10^7^/ml, grown in respective culture medium (18) serving as positive controls. The 12-well plate was then placed in a 37°C incubator with 5% CO_2_ overnight. Negative control wells (A4, B4, and C4) with NHP tissue received culture medium alone without any spirochetes.

On the next day, three of the infected slices from wells B1–B3 and one uninfected slice from the well B4 were transferred into a fresh 12-well plate containing 4% paraformaldehyde (PFA) in PBS (Fisher scientific, AAJ19943K2) and incubated for 24 h at 4°C. Tissue slices were then washed in 1X PBS three times for 1 h each. Later, these fixed tissues were immersed in 10% sucrose solution made in 1X PBS and let to sink to the bottom of the well, which took ~24 h. This step was repeated with 15% sucrose and twice with 30% exchanges. The tissue was then wiped with Kimwipes to remove excess solution and transferred into a cryomold (Usplastic, 75642) containing optimal cutting temperature (OCT) compound (VWR avantor, 25608-930) and were snap-frozen in a dry ice/ethanol bath, then stored at −80°C until further use. For paraffin-embedded blocks, three infected (A1–A3) and one uninfected (A4) tissue slices were embedded in paraffin after the PFA washing step. The rest of the three infected (C1–C3) and one uninfected (C4) tissue slices that were not treated by any fixative were directly embedded in OCT compound and were snap-frozen, serving as fresh-frozen control blocks. Fixation and cryoprotection limit the extent of ice crystal damage to fine histological detail (20).

#### Human Sample Preparation and Assay Design

Brains removed at autopsy were sent, cold but not frozen, to the Neuropathology Laboratory at the New York State Psychiatric Institute. Brain stem and cerebellum were removed from the fresh brain and midsagittal sectioning was performed to separate cerebral hemispheres and cut into 2 cm coronal slices. The right hemisphere slices, brain stem, cerebellum, spinal cord, and the rostral 2 cm slices of the left hemisphere were rapidly frozen, either in 1,1,1,2- tetrafluoroethane or in dry ice/acetone. Frozen slices were placed in individual plastic bags and stored at −80°C. The remaining left hemispheric 2 cm slices were fixed in 10% buffered formalin at room temperature for 5 days, then rinsed and stored at 4°C in PBS with 0.02% sodium azide.

For this study, selected regions were dissected from the frozen slices over dry ice with a dental drill and shipped from New York to New Orleans on dry ice. Immediately after receipt, the frozen slices were embedded in OCT cryomolds as described above and stored at −80°C until further use. Pieces of formalin fixed tissue were sent with cold packs. Paraffin blocks were shipped at ambient temperature.

**Figure 2:**
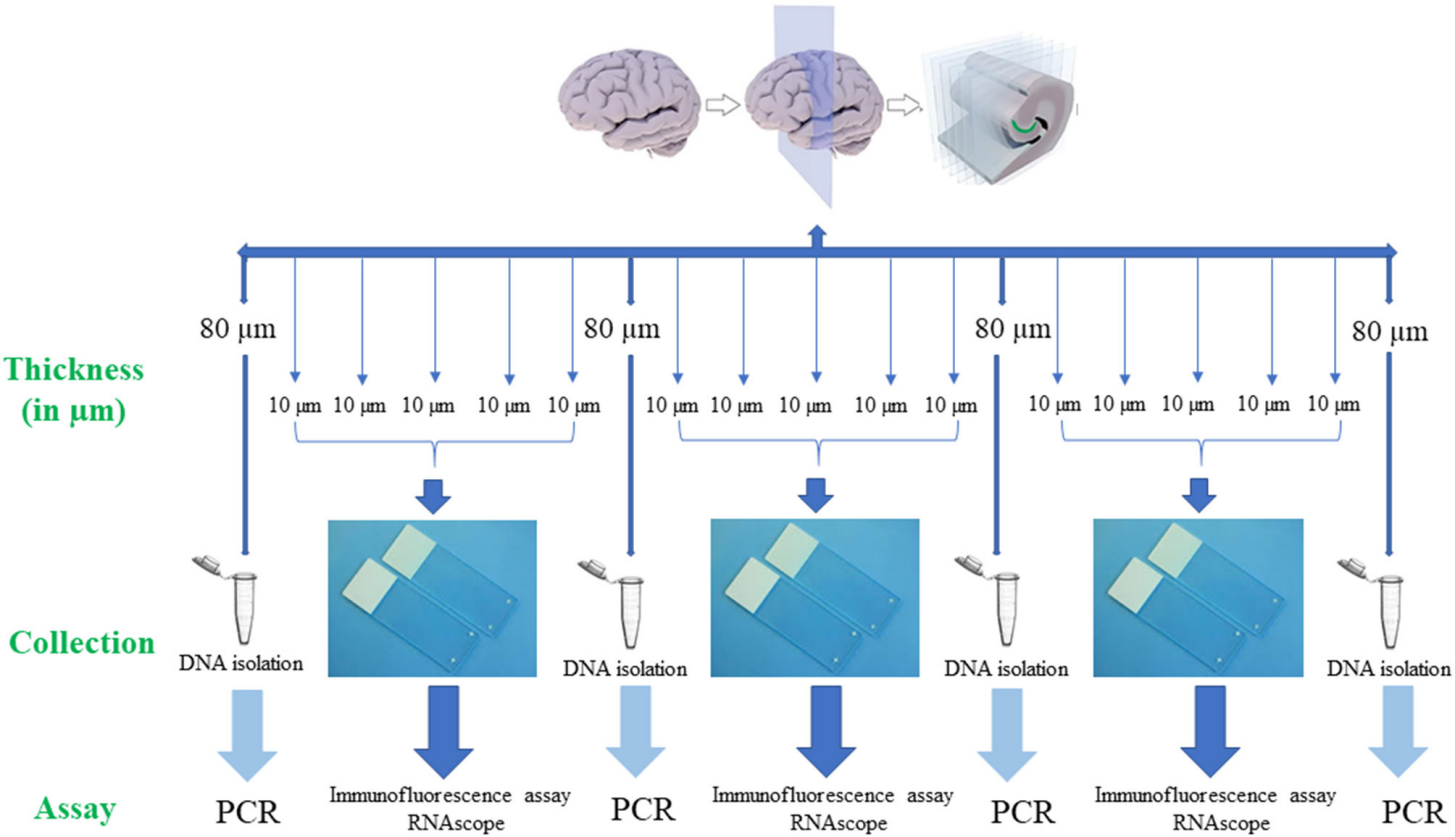
Human brain tissue samples assay design. Serial sections of fresh-frozen samples/fixed tissue samples were analyzed according to the above design. Firstly, 80 μm thickness of the tissue sample was collected into a 1.5 ml microcentrifuge tube for the extraction of the DNA. Next, 5 serial sections each of 10 μm in thickness that were adhered to a positively charged glass slide were used for immunofluorescence (IFA) and RNAscope assays. If these tissue slices were negative for all the assays, subsequent 6 sections, one of 80 μm and five of 10 μm were used for the analysis. This entire process is repeated to a depth of ~500 μm^−1^ mm of the tissue. Once the Borrelial DNA was detected using PCR, the rest of the tissue slices were used for both IFA and RNAscope assays.

For PCR, 80 μm thick sections (Figure 2) were used to extract DNA from the tissue sample. Subsequent 4 serial sections of 10 μm each were used for immunofluorescence assay to detect *B. burgdorferi*. Additionally, one section of 10 μm was stained for RNAscope to determine the integrity of the RNA in the tissue sample. If the tissue sample doesn't express 4–9 dots of PPIB, which is considered a standard to determine the RNA quality of the brain sample, degradation of the RNA in the tissue sample is probable. A total of ~500–1,000 μm thickness of the tissue was analyzed with these multiple methodologies to evaluate the brain tissue samples for borrelia spirochetes.

#### Immunofluorescence Protocol

PFA-treated tissues that were embedded in paraffin were sectioned at 5 μm in thickness and were mounted on positively charged slides (Fisher Scientific). The slides were then allowed to dry at room temperature. After drying, slides were kept in slide boxes and stored in a desiccator that was placed at 4°C until further use. Just before the experiment, sections were deparaffinized using a deparaffinizing station (Leica Autostainer XL) that goes through a series of xylenes followed by graded alcohols. The slides were washed three times with 1X PBS pH 7.4 for 10 min each while gently rocking in a Coplin jar. Deparaffinization was followed by a heat-mediated citrate buffer antigen retrieval (HC-AR) step. For this, slides were immersed in a Coplin jar containing 1X citrate buffer (Vector Labs, H-3300) that was exposed to 5–6 (20 s) cycles of microwave heating until the buffer reached 99°C. Then, the jar with slides was tightly closed and immersed in a water bath maintained at 90°C for 15 min. Later, the jar was left at room temperature for additional 20 min before proceeding to the next step. Slides were then washed and incubated in permeabilization solution containing 0.2% fish skin gelatin (FSG) (Sigma, G704) and 0.1% Triton X-100 (TX-100) made in 1X PBS for 1 h at room temperature under gentle shaking. TX-100 was washed from the slides using PBS/FSG buffer. Slides were then placed in a dark humidifying staining chamber, the tissue sections were circled with a hydrophobic pen and the tissue was covered with blocking buffer (10% Normal Goat Serum-Gibco 16-210-072, in PBS containing 0.2% FSG) for a minimum of 1 h at room temperature. After blocking, tissue sections were incubated with primary antibody rabbit polyclonal anti–*B. burgdorferi* [derived from a hyperimmunized rabbit 6 weeks after inoculation with *in vitro* propagated *B. burgdorferi* strain B31 (21)] for 1 h followed by TX-100 buffer and PBS/FSG washes. Slides were then incubated with secondary antibody Goat Anti-rabbit IgG-Alexa Fluor 488 (Thermo Fisher Scientific, A-11034) diluted 1:500 in blocking buffer. Slides were washed as after incubation with primary antibody, then partially dried by tapping the glass edge onto a paper towel. Finally, to enhance the specificity of the fluorescence signal, a final incubation step with TrueBlack Lipofuscin Autofluorescence Quencher (Biotium, 23007) was performed (22). For this, slides were incubated with 1X TrueBlack solution for 30 s. Only one slide at a time was processed for this step. After 30 s, the quencher was rinsed off of the slides with PBS. Under gentle rocking, slides were then washed three times in PBS (22). Slides were then counterstained with DAPI for 10 min to stain nuclei (EMD millipore, 2160) and mounted with anti-quenching solution (Thermofisher, P36934) and coverslipped. To prevent the decay of fluorescent signal intensity, slides were examined and photographed within 1 week of completion of the staining procedure.

For Fixed-frozen samples, tissues were sectioned at a thickness of 10 μm on a cryostat and mounted onto positively charged slides. These sections were then dried in −20°C freezer overnight. Slides were then individually wrapped in aluminum foil and stored at −80°C until further use. On the day of staining, slides were thawed at room temperature for 20 min followed by three 1X PBS washes each for 10 min each. Then slides were introduced directly into the antigen retrieval solution as just described, and similar steps were followed for the entire staining process. For fresh-frozen tissue blocks, sections were cut at a thickness of 10 μm and stored similarly to fixed-frozen slides. On the day of staining, after PBS washes, slides were immersed in 4% paraformaldehyde for 20 min at 4°C. Slides were then washed three times with 1X PBS followed by permeabilization step and remaining staining process is as described above.

Imaging was performed using a Nikon Ti2-E motorized fluorescence microscope equipped with pco.edge SN:61005789 camera, Plan Apo λ 40× objective with a numerical aperture 0.95 and refractive index 1.000, and a final image with a resolution of 2,048 × 2,044 pixels. Control NHP slides and experimental autopsy tissue slides were imaged during the same session with identical acquisition parameters. Fluorescence intensity was optimized on control tissues to eliminate the tissue background and remained constant for all the experimental slides. When made, adjustments to brightness, contrast, or color balance were applied to the whole image.

#### RNAscope (RNA *in-situ* Hybridization)

RNAscope is commercially available from Advanced Cell Diagnostics, Inc. (ACDBio) (23). RNAscope was performed according to the manufacturer's guidelines from RNAscope Multiplex Fluorescent Detection Kit version 2 (323100-USM). Typically, the probes provided in the kit hybridize to the specific target RNA molecule. These probes contain 20 ZZ probe pairs (50 bp/pair), each pair hybridizes contiguously to the target region, covering ~1,000 bp. Each “ZZ” tail sequence together possess a 28-base hybridization site for the preamplifier, which in turn contains 20 binding sites for the amplifier. These amplifiers contain 20 binding sites for the label probe, yielding a total of 8,000 labels for a target RNA molecule of 1,000 bp (23). The single Z probe binding doesn't affect the signal amplification. The pre-amplifiers won't bind to the single Z probe, preventing the amplification of non-specific signals, contributing to the specificity.

Tissue samples that were positive for both PCR and Immunofluorescence were tested with RNAscope. Three serial tissue 10 μm sections adjacent to the section that was positive for immunofluorescence, were used. These three sections were incubated with hydrogen peroxide at room temperature for 10 min and washed three times with distilled water for 1 min each. Next, slides were immersed in the antigen retrieval solution that was pre-boiled to a temperature of 99°C. slides were left in the solution for 15 min. After the treatment, slides were washed in distilled water for 15 s and were transferred to 100% alcohol and let sit for 3 min. Next, slides were placed at 60°C oven until dried and a hydrophobic barrier was created around the tissue using a pap pen. Once the barrier was completely dried, tissue sections were covered with protease III solution and incubated at 40°C for 30 min in the EZ Hybridization oven (ACD, 321710) using the humidity control tray (ACD, 310012) and slide rack (ACD, 310017). A wet humidifying paper was placed in the humidity control tray to maintain the humidity. After the protease treatment, slides were washed 3 times with distilled water 1 min each. Later, we used a 3-plex positive control probe, a mixture of three endogenous housekeeping probes-UBC (ubiquitin C), PPIB (cyclophilin B), and Polr2A (DNA-directed RNA polymerase II RPB1), each assigned to an individual detection channel, to determine the RNA integrity of the tissue sample on one slide. dapB was used as a negative control probe (ACD, *Bacillus subtilis dapB*, 320871) on a slide and a probe against *Borrelia burgdorferi* (ACD, 23S rRNA transcript, 468211) was added to an individual slide and incubated in the hybridization over as mentioned above for 2 hrs. After probe hybridization, amplification and detection steps were performed. Signal amplification is a branched hybridization of amplifiers that are unique to each individual channel. Amplifier 1 was initially incubated on slides for 30 min at 40°C and washed with wash buffer three times each for 2 min. Slides were then incubated with Amplifier 2 for 30 min as before, washed, and then incubated with a final layer of amplifier, Amplifier 3, and incubated for 15 min at 40°C and washed as before. For development of the signal, HRP (horseradish peroxidase)-C1 was added to the tissue to target the probe assigned to channel C1 and incubated in the hybridization oven for 15 min at 40°C. Finally, slides were incubated with fluorescent molecules from PerkinElmer [Tyramide Signal Amplification (TSA) plus fluorescein: NEL745001KT or TSA plus Cyanine 3 system: NEL744001KT or TSA plus Cyanine 5 system: NEL745001KT]. These fluorescent molecules bind to the cascade of signal amplification molecules resulting in the signal detection. Similarly, HRP-C2 and HRP-C3 were incubated on the tissue for the signal development of probes targeted on channel 2 and channel 3. Individual channels were assigned different fluorophores.

#### Conventional and Nested Polymerase Chain Reaction (PCR)

Genomic DNA from the tissue samples was extracted using QIAamp DNA FFPE kit (Qiagen, 56404). Initially, slides were deparaffinized using Leica Autostainer X and tissue sections were scrapped into a sterile microcentrifuge tube using sterile blades. For each preparation, tissue sections with a thickness of 80 μm and a surface area of up to 250 mm^2^ was combined in a tube. Usage of the tissue section beyond this recommendation leads to clogging of the purification column. The tissue sections in the tube were then resuspended in a mixture containing 180 μl of ATL and 20 μl of proteinase K and incubated at 56°C for 1 h to overnight until the liquid in the tube appeared clear. The microcentrifuge tube was then incubated at 90°C for 1 h to reverse the aldehyde cross-linking. After this step, 200 μl of AL buffer was added and mixed thoroughly by vortexing, and an additional 200 μl of (96–100%) ethanol was added and mixed. The lysed samples were then loaded onto a QIAamp minElute column and centrifuged for 1 min. The columns were then placed in a new collection tube. Next, two column wash steps were performed to remove any unwanted material. The column was then placed in a new collection tube and centrifuged at 14,000 rpm for 2 min to remove any residual buffer present in the column and then was dried. Finally, the dry column was placed in a new sterile microcentrifuge tube and incubated with 50 μl of pre-warmed (37°C) nuclease-free water for 5 min and centrifuged at full speed for the elution. The isolated pure DNA was quantified using a Nanodrop 2000 spectrophotometer (Thermo scientific).

**Figure 3:**
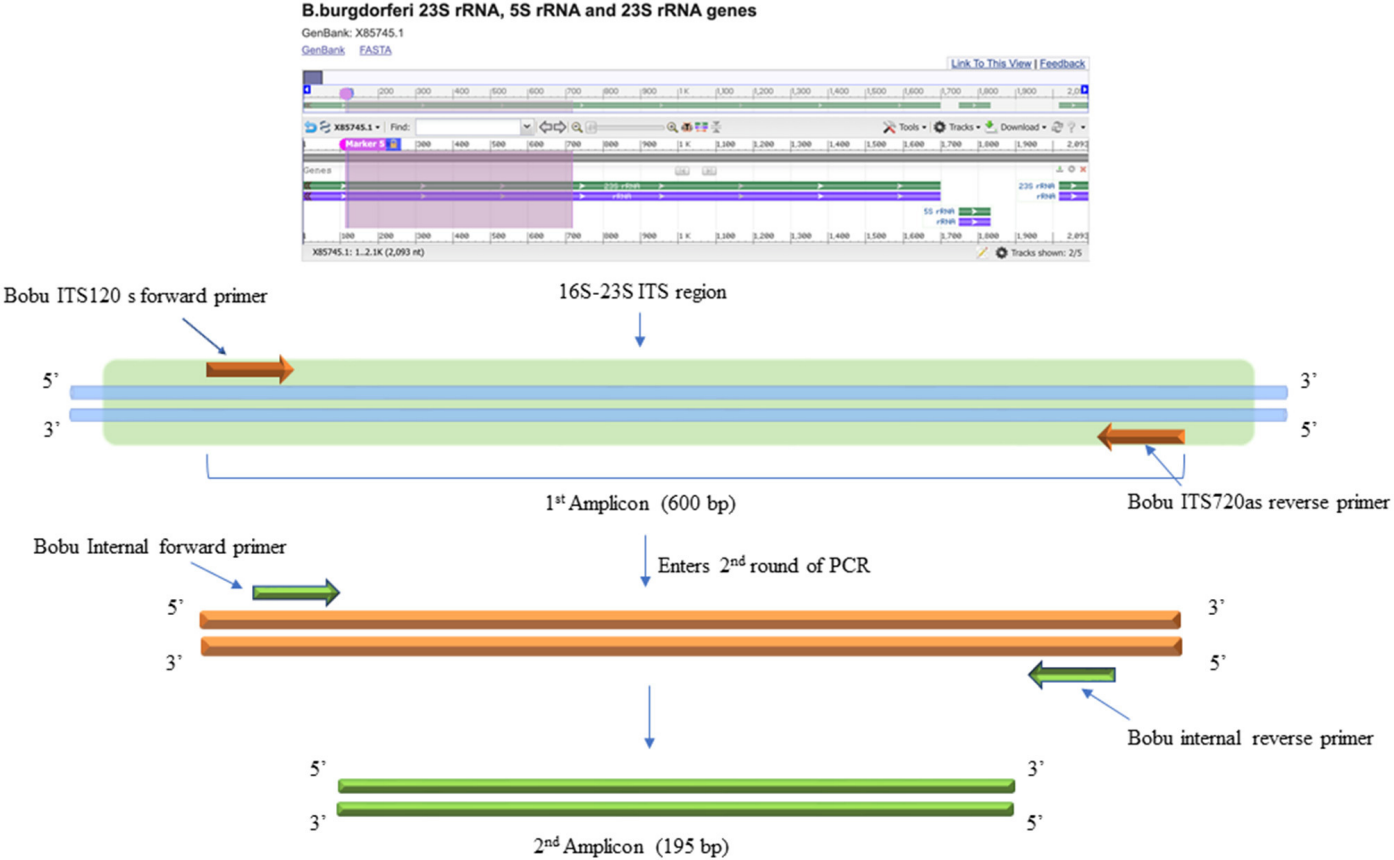
DNA Primers targeting the 16S−23S internal transcribed spacer (ITS) region of *Borrelia burgdorferi* B31 strain. Bobu ITS120 s forward and Bobu ITS720as reverse primers were targeting the region of ITS of the Borrelia genomic sequence with the accession number X85745.1 from 120 to 720 bp making the first amplicon of 600 bp. These primers were designed and tested previously on *Ixodes affinis* and *Ixodes scapularies* tick species that are widely found in coastal plain region of North Carolina. To increase the specificity and sensitivity, nested primers were designed. Bobu Internal forward and Bobu internal reverse primers amplify the initial amplicon to generate a second amplicon size of 195 bp.

The DNA samples were initially tested with standard PCR using primers designed to amplify *Borrelia burgdorferi* 16S−23S ITS region of ribosomal DNA (24). For this, Q5 Hot start High-Fidelity DNA polymerase (NEB, M0493L) was used. Initial PCR was performed using a total of 300 ng of isolated DNA as a template. Negative control included the DNA extracted from uninfected NHP brain tissue and DNA from *B. burgdorferi* incubated NHP brain tissue was used as positive control. The primers used for the initial conventional PCR were F: Bobu ITS120 s 5′ AGGTCATTTTGGGGGTTTAGCTCAGTTGGCT 3′ and R: Bobu ITS720 as 5′ AGTGTCGGGCAAATCCAAACTGAAATCTG 3′ (24). The thermocycling conditions were: Initial denaturation 98°C for 30 sec, followed by 55 cycles of 98°C/10 s, 65°C/15 s, 72°C/18 s and a final extension of 72 C for 2 min. The second round of PCR was performed similar to the first reaction except that, two different primers that are internal to the *Borrelia burgdorferi* 16S−23S ITS region were used to increase the sensitivity and specificity of the PCR (Figure 3). For this, 2 μl of the first PCR product was used as template DNA. Primers used were F: 5′ATTAAGAAAAATGTCTAGA AGCAAAAGCAAGCT3′ and R: 5′TACAATACTTGTCCTTCTCTCAGACATCA3′. Reactions were setup as before. The thermocycling condition were initial denaturation 55 cycles of denaturation 98°C for 30 s, followed by 55 cycles of 98°C/10 s, 55°C/15 s, and 7 s @ 72°C/7 s and a final extension of 72°C for 2 min. For negative and positive controls, 2 μl of the initial reaction was used.

### Case Study Description

This 69 year old woman (Patient 12,577) contracted Lyme disease at age 54 with a well-documented erythema migrans rash accompanied by a severe headache, joint pains and a fever of 104; convalescent serologies were positive on ELISA and both IgM and IgG Western blots. Treatment with doxycycline for 10 days led to symptom resolution. Two years later, a sleep behavior disorder emerged. Four years later, cognitive problems (processing speed, mental tracking, and word-finding) emerged and gradually worsened. Other symptoms included photophobia, paresthesias, fasciculations, and myoclonic jerks. Neurocognitive testing revealed deficits in visuospatial skills and executive functions with preservation of verbal skills, suggesting a neurodegenerative process. Brain Magnetic Resonance Imaging with and without contrast showed mild atrophy and non-specific scattered white matter hyperintensities without enhancement. Brain Single Photon Emission Computed Tomography scans showed decreased perfusion in the right posterior parietal and temporal lobes. Serum was negative or normal for erythrocyte sedimentation rate, c-reactive protein, antinuclear antibody, and thyroid stimulating hormone. PCR assays of blood for *Bartonella henselae, Babesia microti*, and *Borrelia burgdorferi* were negative. Serum C6 ELISA was negative but Lyme IgG Western blot was positive with 9/10 bands. Treatment with IV ceftriaxone at age 60 for 8 weeks led to 60% improvement in cognition and interpersonal engagement; oral amoxicillin 500 mg three times daily was continued for 6 months after the IV treatment. The initial improvement was not sustained and subsequent antibiotic therapy with minocycline was of no clear benefit; gradually her visual spatial skills and executive functions deteriorated further, and anxiety worsened. Serum IgG Western blot continued to be positive. At age 62, a cerebrospinal fluid study demonstrated 4 CSF IgG bands on Lyme Western blot; unfortunately, because CSF and serum ELISA studies were not conducted, intrathecal Bb specific antibody production could not be assessed. Other CSF studies were unremarkable including absence of pleocytosis or elevated protein, absence of P-tau elevation, Venereal Disease Research Laboratory assay, Acid-Fast bacteria, fungi, and negative Herpes Simplex Virus and Epstein-Barr Virus PCRs. A second brain MRI showed periventricular and subcortical T2 hyperintensities possibly due to “small vessel ischemia or demyelinating disorders like Lyme disease.” Fluorodeoxyglucose-Positron Emission Tomography scan showed “diffuse cortical hypometabolism, worse in the posterior parietal and temporal lobes, with sparing of the sensory motor cortex and visual cortex bilaterally—findings consistent with Alzheimer's disease.” The extensive workup at that time led to the diagnoses of both a REM behavioral disorder with verbalizations and movements and a neurodegenerative dementia characterized by expressive aphasia, visual agnosia, anomia, deficits in executive function and calculation, and mild memory problems. Eventually, she developed severe oral dystonia, making feeding progressively more difficult; she died 15 years after the initial infection with *B. burgdorferi*. Early and severe movement disorders, REM behavioral disorder, paranoia, and personality changes all favored a clinical diagnosis of dementia with Lewy bodies.

### Human Control Tissues

Tissue blocks from various regions of seven specimens from brains of deceased Macedonian residents that were housed in the Macedonian/New York State Psychiatric Institute Brain Collection were used as controls. Though none had a clinical history of Lyme disease based on interview with the surviving family members, Borrelia is endemic in Macedonia. These brain tissues were probed in the same manner as the human case study with IFA and PCR-based detection methods.

## Results

### The Case Study Pathology Is Characteristic of Dementia With Lewy Bodies (DLB)

**Figure 4:**
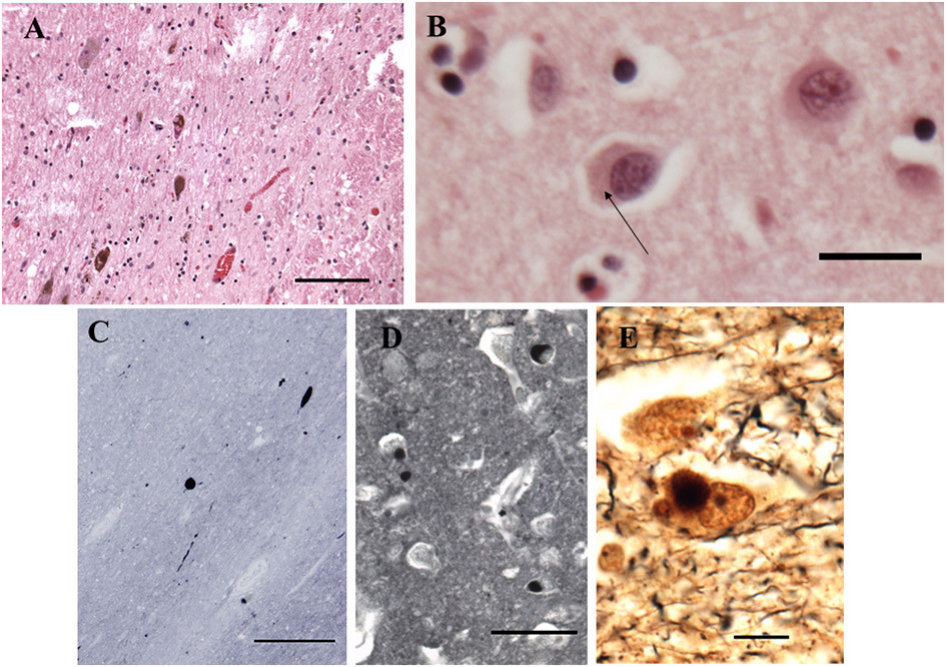
Histopathological findings from the case study. **(A)** Substantia Nigra. H&E. Disintegration of pigmented neurons, with Lewy bodies in two of those remaining (center and lower left). Bar = 100 microns. **(B)** Frontal cortex. H&E. A Lewy body is seen in the central neuron as a perinuclear region of increased eosinophilia (arrow). Bar = 20 microns. **(C–E)** Immunohistochemistry for α-synuclein. **(C)** Substantia nigra pars compacta. Inclusions are seen in cell bodies and neurites. Bar= 200 microns. **(D)** Hilus (CA3) of hippocampus. Bar = 50 microns. **(E)** Bielschowsky stain, hippocampus. Central neuron contains a Lewy body. Bar = 10 microns.

**Figure 5:**
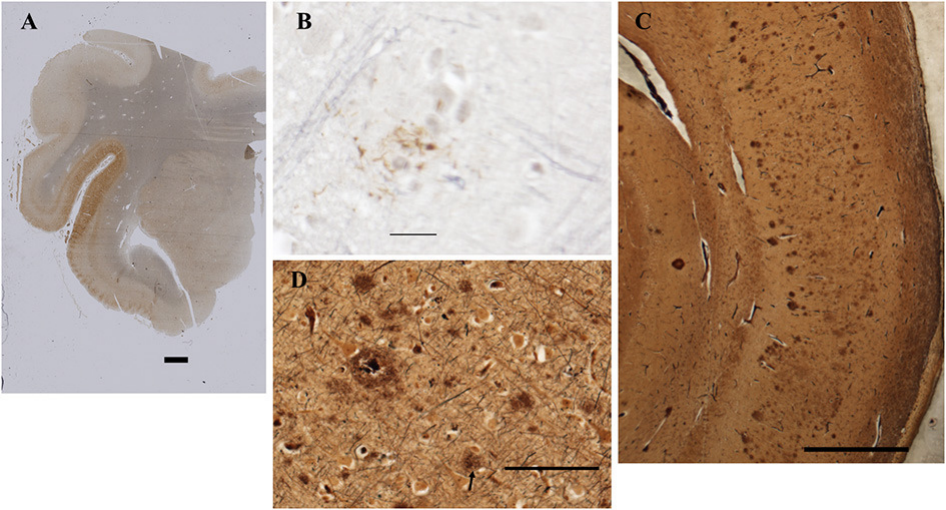
Detection of amyloid plaques in the patient's CNS. **(A)** Amygdala and rostral hippocampus, entorhinal cortex, and transentorhinal cortex. AT8 immunohistochemistry with Verhoeff myelin counterstain. BAR = 2 mm. **(B)** Rare cortical neuritic plaque in middle frontal gyrus. AT8 with Verhoeff myelin counterstain. BAR = 20 microns. **(C)** Bielschowsky stain of CA1 and subiculum demonstrates numerous amyloid plaques. Bar = 1 mm. **(D)** Bielschowsky stain of frontal cortex. Amyloid plaques predominate; rare neuritic plaques are also present (arrow). Bar = 100 microns.

**Figure 6:**
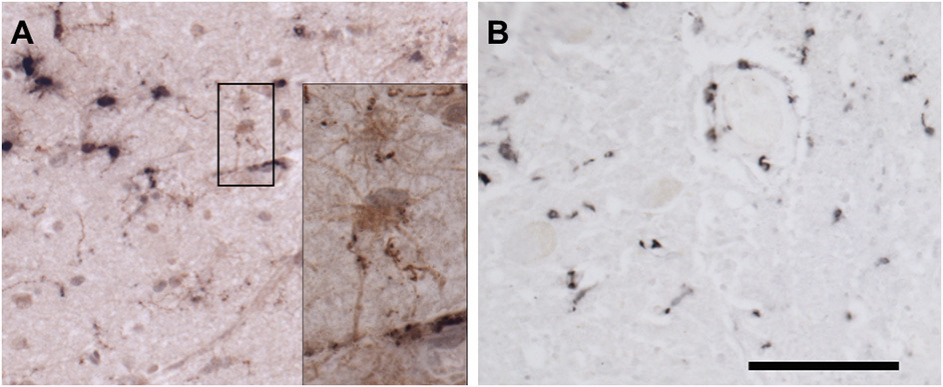
Immunohistochemical staining for microglia. **(A)** Frontal cortex stained for Iba-1 (brown) and CD68 (black). Most microglia in this field contain large deposits of CD68 immunoreactivity and shirt, thick processes, indicating activation. By contrast, occasional surveilling microglia, are also present. The black box surrounds two such cells, whose somas and long, fine processes are apparent in the inset. **(B)** Spinal cord stained for CD163. CD163 is expressed by resident perivascular macrophages, which are normally present, but also by macrophages derived from circulating monocytes. Bar = 100 microns for **(A,B)**. Inset is photographed at three times greater magnification in a stack spanning the thickness of the tissue at 0.25-micron intervals. The “Deep Focus” feature of Neurolucida 360 (MBF Bioscience) was used to combine the best focused objects from each layer of the stack into a single layer.

The fresh brain weighed 996 g and appeared atrophic Coronal sections through the left cerebral hemisphere and brain stem revealed mild enlargement of the lateral ventricle, particularly the temporal horn. The substantia nigra was normally pigmented or nearly so. Microscopically, nigral and cortical Lewy bodies, were seen with hematoxylin and eosin stain (H&E, Figures 4A,B). Immunohistochemistry (IHC) for α-synuclein (clone 42, BD Transduction Laboratories) showed numerous immunoreactive Lewy bodies and fibers in substantia nigra, hippocampal formation and neocortex, Figures 4C–E). IHC for hyperphosphorylated tau (monoclonal antibody AT8; ThermoFisher) revealed intense staining of many limbic neurofibrillary tangles and neuropil threads (Braak stage 2–3, Figure 5), and of occasional neurofibrillary tangles in neocortex, but senile plaques were extremely rare, and each contained only a few fibrils (Figure 5). H&E showed prominent thickening of small blood vessels in gray and white matter, extensive mineralization of pallidal vessels, and rare microglial nodules in the hippocampal formation. Immunohistochemistry for Iba-1 (Wako), CD68 (clone KP1, Dako), and CD163 (clone EDHu-1; Bio-Rad) showed moderate numbers of activated microglia and large numbers of macrophages in hippocampal formation and spinal cord (Figure 6). In summary, we see DLB accompanied by features of Alzheimer's disease, a common presentation.

### Nested PCR Provides Sensitive Detection for *Borrelia* sp. DNA

To determine the presence of *Borrelia burgdorferi*, we first investigated the presence of DNA in the brain samples. To this end, nested PCR was adapted to increase the sensitivity and specificity of the assay (Supplementary Table 1). DNA isolated from frontal cortex of an uninfected non-human primate (NHP) was used as a negative control and as a positive control, DNA isolated from NHP frontal cortex tissue that was incubated with *B. burgdorferi* spirochetes was used (Supplementary Figure 1). To prevent cross-contamination between positive control and autopsy specimens, DNA from autopsy tissue samples was isolated in laboratories that were never exposed to *B. burgdorferi* DNA.

### Immunofluorescent Detection Is the Most Reliable Method to Detect Spirochetes in Fixed Tissues

**Figure 7:**
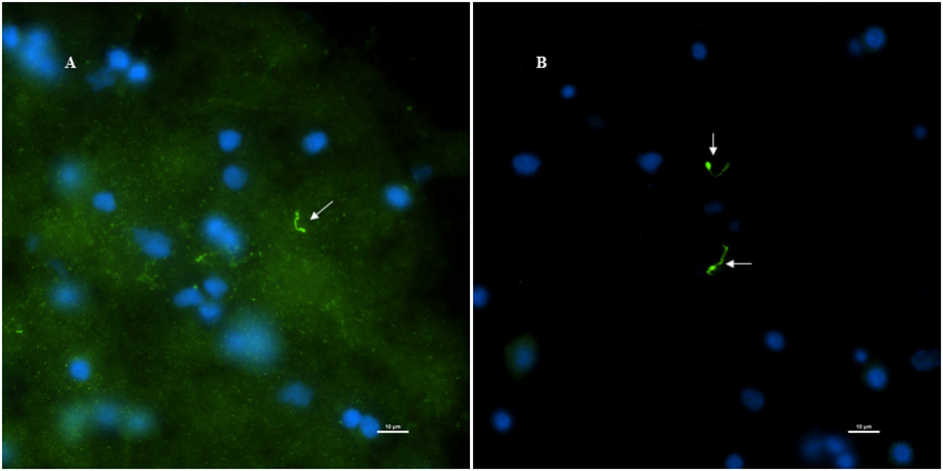
Non-human primate (MJ 61) frontal cortex tissue was incubated with *Borrelia burgdorferi ex-vivo*. These *ex-vivo* tissues were fixed, sectioned at 10 μm, and stained against Borrelial spirochetes. **(A,B)** Are representative images of positive staining of Borrelial spirochetes. White arrows were pointed toward Borrelial spirochetes that were stained with primary rabbit polyclonal anti-Borrelia antibody and secondary Goat anti-rabbit IgG antibody conjugated to Alexafluor 488. To increase the signal intensity, **(B)** was incubated with Autofluorescence quencher after completion of immunofluorescence that caused a significant decrease in the autofluorescence caused by lipofuscin and aldehyde fixation. Scale bars−10 μm. Image was acquired with a Nikon fluorescence microscope using a 40x objective.

To strengthen the results of our PCR, we next explored immunofluorescence assay (IFA) to see if these tissue specimens contain morphologically intact spirochetes. Initially, we tested our rabbit polyclonal antibody on NHP frontal cortex tissue that was incubated with borrelial spirochetes to see the specificity of the antibody (Figure 7A). The image cleanliness was increased after incubating the tissue with the lipofuscin quencher that quenches the fluorescence of lipofuscin aggregates, which are formed during normal aging process (Figure 7B). Secondary antibodies were tested with isotype controls for specificity on human (Supplementary Figure 2A) and macaque (Supplementary Figure 2B). Optimal fixation time is very crucial for the success of the IFA. Tissues must be properly fixed; under-fixation leads to tissue deformation, shrinkage and autolysis (25), whereas over-fixation may permanently mask the antigen, abolishing its detection even after antigen-retrieval steps (26).

### RNAscope Is a Sensitive Technique for Finding Viable Pathogen but Requires Freshly Prepared Tissue

**Figure 8:**
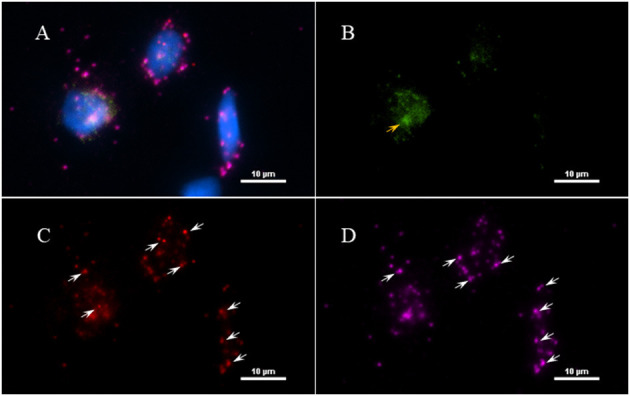
Validation of RNA integrity on Fresh frozen NHP (MJ 61) frontal cortex brain tissue using RNAscope assay. NHP tissue was stained with a 3-plex positive control probe POLR2A targeting DNA-directed RNA polymerase II RPB1, PPIB targeting cyclophilin B, and UBC that targets Ubiquitin C. In terms of relative expression levels, UBC is highest, PPIB is considered a moderate-high, POLR2A is moderate-to low expressor target. Successful staining has 4–9 dots per cell of PPIB/POLR2A and UBC of 10–15 dots per cell. PPIB is considered a standard to determine the RNA quality of brain samples. **(C)** Represents red fluorescence, indicating the specificity of PPIB, whereas, **(D)** displays a far-red puncta targeting UBC gene. Absence of POLR2A signal in **(B)** represents, quality of RNA being partially compromised. A merge image of all the channels can be seen in **(A)**. White arrows were pointing toward the fluorescence puncta and orange arrows toward autofluorescence of the tissue.

**Figure 9:**
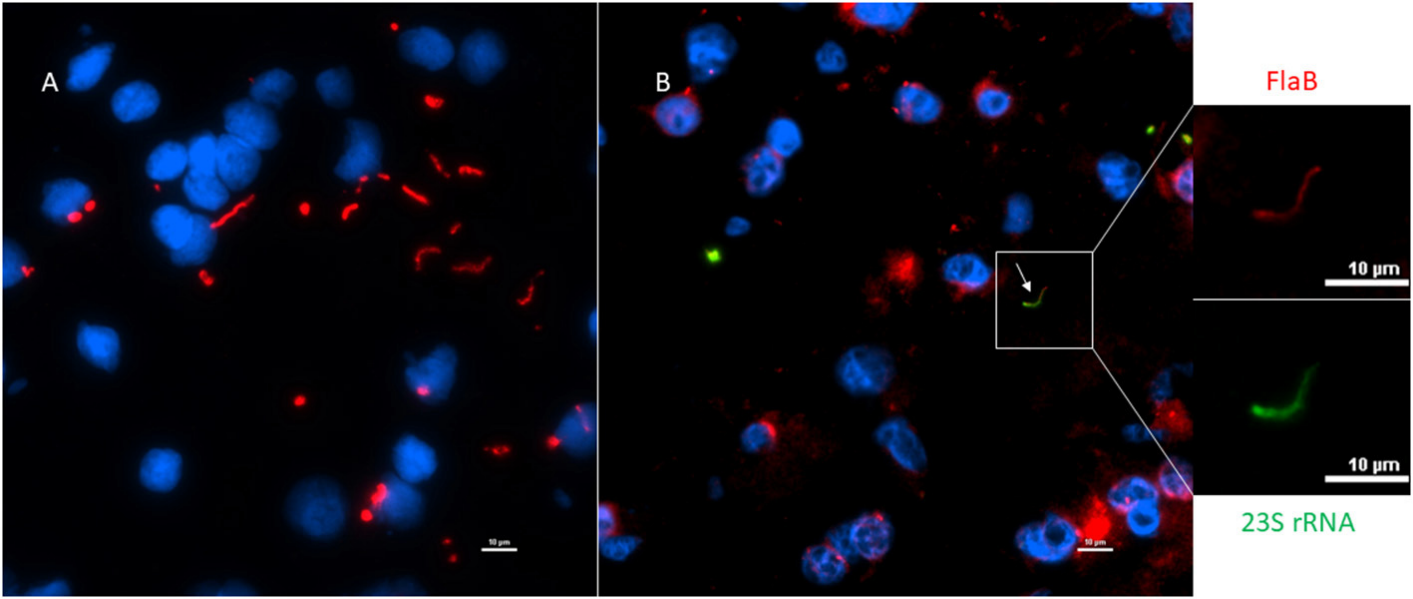
Technical validation of RNAscope assay performed on NHP (MJ 61) frontal cortex brain tissue cultured with *Borrelia burgdorferi*. **(A)** Fixed-frozen tissue: Spirochetes are highlighted by an intense red (cy3, PerkinElmer catalog number NEL744001KT), indicating strong specificity of *B. burgdorferi* probe (*B. burgdorferi* probe targeting the 23S rRNA transcript; Advanced Cell Diagnostics catalog number 468211). **(B)** Fresh-frozen tissue: RNAscope assay combined with Immunofluorescence (IFA). Initially, spirochetes were probed with 23S rRNA and detected using fluorescein fluorophore (PerkinElmer catalog number NEL741001KT). To counterstain the spirochetes with IFA, tissue was incubated with primary anti-FlaB mouse monoclonal antibody H9724 and goat anti-mouse IgG2a Alexa Fluor 594 (Thermofisher catalog number A-21135). White arrow indicates an intact spirochete showing the colocalization of RNAscope probe with monoclonal antibody with subsequent green and red fluorescence.

**Figure 10:**
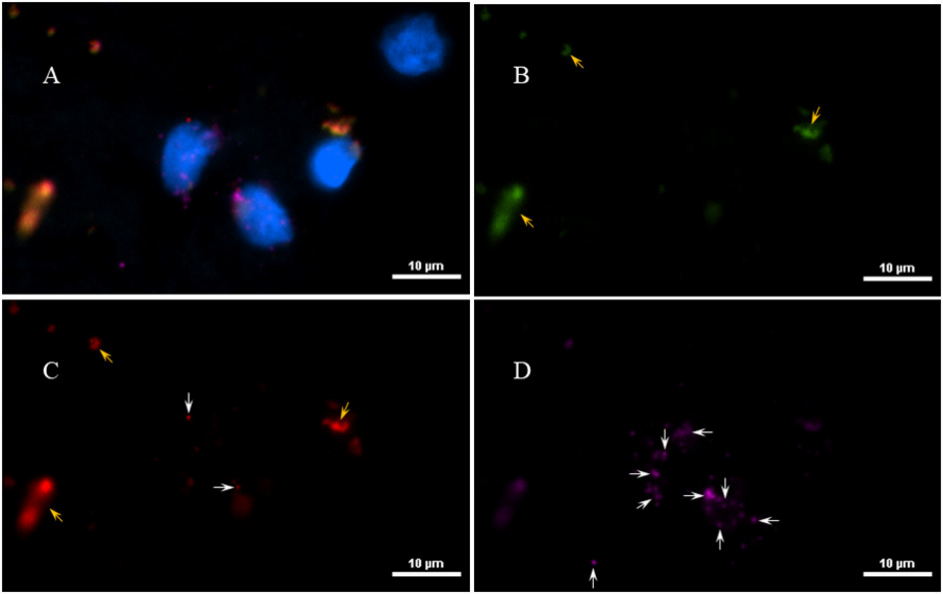
Validation of RNA integrity on a fresh-frozen tissue sample from patient 12,577 autopsy tissue using RNAscope assay; **(A)** = merged. Tissue was stained with a 3-plex positive control probe POLR2A targeting DNA-directed RNA polymerase II RPB1, PPIB targeting cyclophilin B, and UBC that targets Ubiquitin C **(D)**. Absence of signal in POLR2A **(B)** and only 2 puncta of PPIB per cell **(C)** indicates that the quality of the RNA has been compromised. White arrows were pointing toward the fluorescence puncta and orange arrows toward autofluorescence of the tissue.

Next, to determine if the persistent Borrelia spirochete was actively dividing, we probed the tissue with RNA probes from ACDBio. First, to optimize the protocol, the probes were tested on NHP control tissue (Supplementary Figure 3). To determine the RNA integrity of the NHP tissue, 3-plex positive control RNA probe was used on NHP tissue. Figure 8 shows the staining of frontal cortex region of NHP fresh frozen tissue, where, white arrows in Figures 8C,D were pointed toward the positively stained PPIB dots and UBC dots, respectively. PPIB is considered a standard to determine the RNA quality of the brain samples. However, the low expresser target POLR2A was not detected in these tissues indicating partial degradation of RNA. The reason behind this could be that the tissues were processed after 16 h of incubation with the Borrelia spirochetes. Next, to test the 23S rRNA probe that targets the Borrelial RNA, the frontal cortex region of NHP fresh frozen tissue and fixed tissue that were incubated with Borrelia spirochetes was used. Red fluorescence in Figure 9A represents Borrelia spirochetes in NHP fixed tissue. Whereas, tissue in Figure 9B is a representative image obtained by combining RNAscope with the immunofluorescence assay. Initially, the tissue was probed with the 23S rRNA probe and detected using fluorescein fluorophore. Next, the same tissue was counterstained for IFA assay using the borrelia specific anti-FlaB monoclonal antibody H9724 (27, 28) and Goat anti-mouse IgG2a secondary antibody conjugated to Alexa Fluor 594. The inset in Figure 9B showing an intact spirochete that is labeled green with RNAscope assay and red with IFA assay. After optimizing the positive control probes and Borrelia specific RNA probes on NHP tissues, the positive control probe was used to determine the integrity of RNA in human autopsy samples. For this, fresh-frozen tissue was used. Figure 10 is a representative image of the positive control probe showing only 5–6 dots of UBC and one dot of PPIB per cell indicating that the RNA integrity of the sample has been declined and failed QC for RNAscope.

#### The Patient Harbored Borrelia in the Amygdala and Spinal Cord

**Figure 11:**
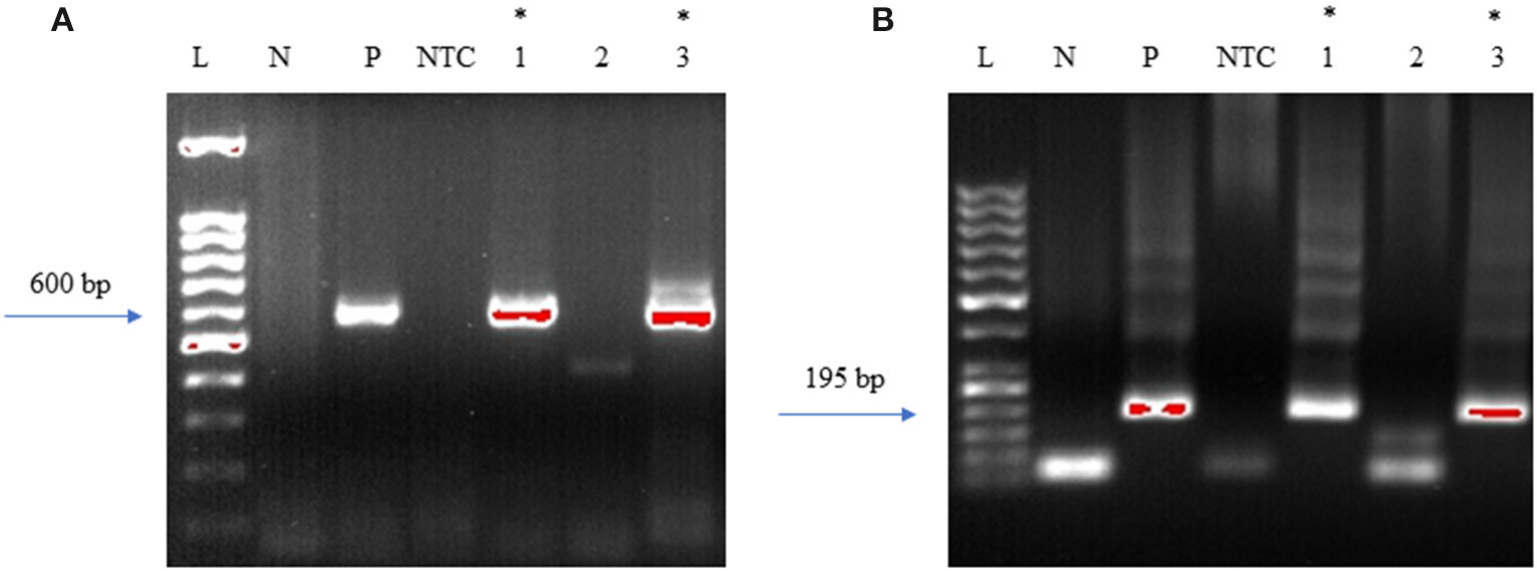
Primers amplify Borrelia 16S−23S internal transcribed spacer (ITS) region. Bobu primers generate an amplicon size of 600 bp. To increase the specificity Bobu internal primers were designed to generate an amplicon size of 195 bp. **(A)** External PCR: L-molecular weight marker of 100 bp, N-negative control, P-positive control, NTC-No Template Control, 1–12,577 amygdala, 2–12,577 pons, 3–12,577 spinal cord. **(B)** Internal PCR: L-molecular weight marker of 50 bp, N-negative control, P-positive control, NTC-No Template Control, 1–12,577 amygdala, 2–12,577 pons, 3–12,577 spinal cord. *Samples that were positive to Borrelial DNA.

Initially, the first round of PCR was done on the DNA extracted from fresh-frozen tissues of amygdala, pons, and spinal cord, using primers that were previously used to target the 16S−23S internal transcribed spacer (ITS) DNA region of Borrelia found in *Ixodes affinis* and *Ixodes scapularis* (24). Out of the three tissue regions that were tested for PCR, amygdala and spinal cord were PCR positive for Borrelia DNA (Figure 11A). This initial amplicon of 600 bp was used for the second round of PCR using primers that were designed and tested in-house. For this, 2 μl of the initial reaction was used as a template DNA. As expected, amygdala and spinal cord tissues were positive with the nested PCR (Figure 11B). To avoid cross-contamination during reaction setup, a no template control was included. Amplicons from all three of the positive PCR reactions were sequenced and the results are shown in Supplementary Table 3. According to the BLAST, these sequences were aligned with 98–99% identity with a query coverage in between 48 and 62% to different isolates of *B. burgdorferi*. Our results demonstrated specificity with the nested primers and some amplification from human control tissue. Other than the case study, one tissue from seven Macedonian control autopsy specimens was positive by both primer sets (Supplementary Figure 4 and Supplementary Table 2). As mentioned, Borrelia infection is endemic in the region, so some of those individuals may have harbored an asymptomatic or unrecognized persistent infection. Immunofluorescent staining of the control patient tissue did not reveal obvious spirochetes.

**Figure 12:**
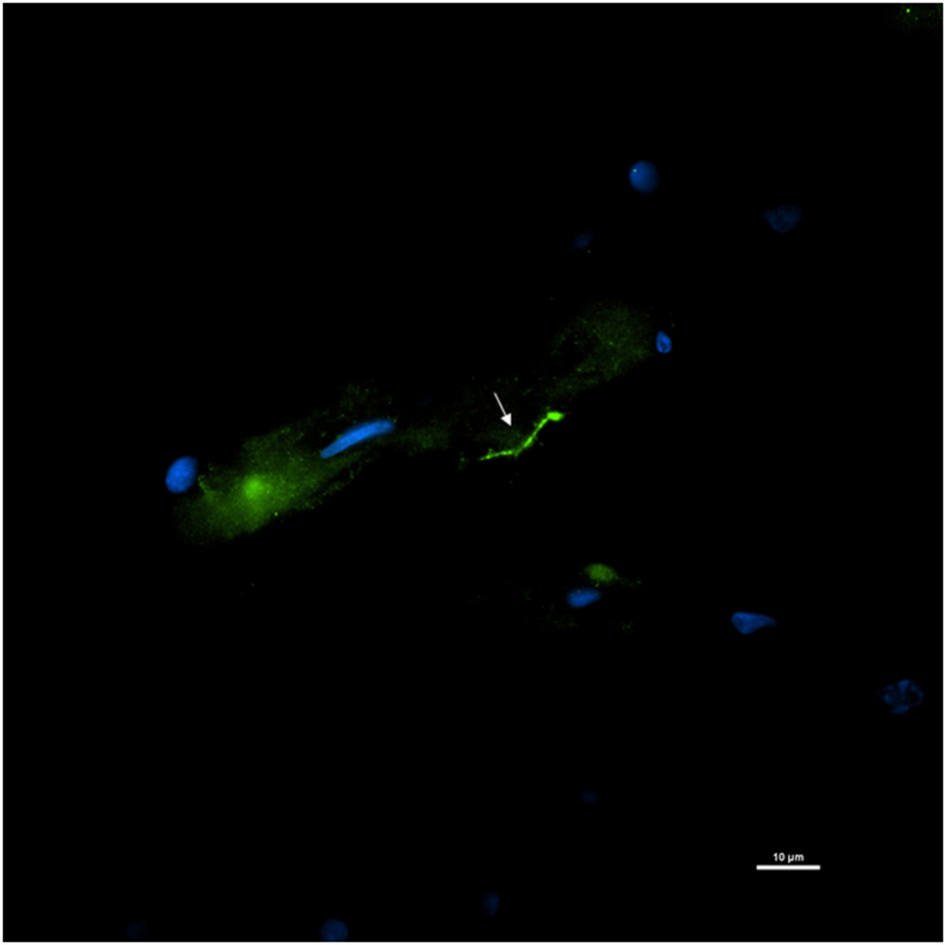
Detection of an intact Borrelia spirochete in a fixed autopsy tissue using Immunofluorescence assay. 12,577-spinal cord autopsy tissue was immunostained with rabbit *B. burgdorferi* hyperimmunized polyclonal (primary) and goat anti-rabbit IgG Alexa Fluor 488 (secondary) antibodies. White arrow points toward a green fluorescence of morphologically intact spirochete. Scale bar−10 μm. Image was acquired with a Nikon fluorescence microscope using a 40x objective.

Tissue slices adjacent to the autopsy tissue specimens that were positive for DNA were stained for the presence of persisters. For this, slides were stained with the primary rabbit polyclonal anti–*B. burgdorferi* antibody and a goat anti-rabbit IgG-Alexa Fluor 488 secondary antibody. An intact Borrelia spirochete with the expected morphology was identified within fixed tissue of the spinal cord (Figure 12). The spirochete appeared to be adjacent to vasculature.

Next, RNAscope was tested on serial sections of the tissue where the persistent spirochete was detected by IFA. However, positive staining of the Borrelia with 23S rRNA probe was not detected. Absence of the concurrent RNA staining could be due to multiple factors, the most prominent of which being delayed sample collection after the death. According to the manufacturer's instructions, the post-mortem tissue sample should be collected within 12 h after the death to preserve the RNA integrity and tissues collected after 24 h would lend decreased sensitivity. Overfixation of the tissue may also decrease detection sensitivity. The optimal fixation of the autopsy tissue specimen would be between 16 and 32 h in freshly prepared 10% neutral buffered formalin solution or 4% PFA at room temperature. However, thick brain slices require a longer period of fixation. Considering the low copy number of the Borrelia transcripts present in the autopsy specimens, this assay would be highly dependent on tissue preparation techniques to preserve RNA integrity.

## Discussion

Two reasons exist for the interrogation of autopsy specimens for the Lyme disease spirochete. First, in patients with a known history of Lyme disease and a record of antibiotic treatment, the potential for treatment to fail in eradicating the infection can be evaluated. Notably, a detailed patient history, including history of possible second *B. burgdorferi* infection and treatment non-compliance, is necessary. Given the difficulty in recovering organisms from living people, looking at post-mortem tissue can provide some resolution on the issue of persistence. Secondly, patients such as the one presented here, can manifest neurological disease that may or may not be related to infection. Here, the patient developed dementia with Lewy body pathology. While availability of tissue may be a challenge, the role of *Borrelia burgdorferi* in the etiology of chronic neurological disease, can be studied as a “proof of principle.”

Our study confirms that *Borrelia burgdorferi* was detected in the brain and spinal cord tissue of this patient with a history of previously treated Lyme disease. These results however do not clarify whether the Borrelia infection had anything to do with her progressive neurodegenerative disorder. It is possible this is an unrelated incidental finding or that there is a relationship between CNS infection with Bb and the development of a neurodegenerative dementing disorder.

Previous studies suggest that Borrelial spirochetes can start invading the nervous system during early stages of the infection resulting in meningeal seeding (29), and this later leads to neuroborreliosis. To define the pivotal neurological deficits, a study in Europe examined the clinical manifestations of 68 patients hospitalized for neuroborreliosis. Meningitis was found to be one of the least frequent conditions, present in 6% of the patients (30), whereas cranial neuritis was the most frequent (25%). The clinical Lyme case presented here was documented with meningismus at the time of the EM rash, supporting the possibility of mild meningitis at early infection. Bacterial meningitis leading to cognitive impairment was well-studied in *Treponema pallidum* in relation to dementia (31). *B. burgdorferi* infection has also been associated with mild (32) to severe (33) cognitive deficits. In the endemic areas of Lyme disease, Borrelia infections as a possible cause of cognitive impairment has to be carefully considered.

Neurotropic viruses have been associated with neurodegenerative syndromes, as have spirochetal infections (34–38). Precedence for an association between *B. burgdorferi* infection, specifically, and dementia exist (38–42), however there are also reports that have failed to link *B. burgdorferi* to AD (43). Evidence that amyloid plaques may have a functional protective role in combatting microbial infection has also come to the fore (44). Evidence that Borrelia can induce amyloid production is suggestive of a possible mechanism for development of AD (45–47).

To comprehensively evaluate the possible role of Borrelia in dementia (Alzheimer's and LB), 20 patients were identified from a total of 1,594 patients who were seen for dementia, who had positive intrathecal anti-Borrelia antibody index (AI), indicative of past or present Lyme disease (48). Among these 20 patients, 7 patients with neuroborreliosis dementia showed stability or mild improvement in their cognitive functions after treatment with ceftriaxone, and the others showed progressive worsening despite antibiotic treatment (48). The individual in our clinical case reported 60% cognitive improvement after the antibiotic treatment. However, this improvement was not sustained and cognition gradually worsened, a finding consistent with a previous study demonstrating cognitive functional deficits in treated Lyme neuroborreliosis patients (49). The possible anti-inflammatory effects of antibiotic cannot be discounted (50).

A recent study aimed at testing the hypothesis that polymicrobial infections contribute to Alzheimer's disease was conducted. Brain sample tissues were probed for *B. burgdorferi* using a commercially available monoclonal antibody (43). However, this study was unable to demonstrate the presence of Borrelia spirochetes in the tissue samples. The possibility exists that this could be due to the selection of antibody. The polyclonal used exhibited some cross-reactivity to fungal structures and the monoclonal antibody may have targeted an antigen (OspA) that is downregulated as spirochetes migrate from tick to mammalian host. Studies have shown that the expression of the OspA is abundant on the surface of bacteria when residing in tick midguts, but its expression is repressed during host infections (51). However, there are studies showing the expression of OspA in one-third of the spirochetes inoculated in mice and in cerebrospinal fluid of early neurologic Lyme disease (52, 53), suggesting that OspA might not be an ideal choice in the interpretation of the analysis of a study. In a recent study from our laboratory, we were able to identify *B. burgdorferi* with a monoclonal antibody to OspA in some tissues (e.g., heart) but not others, where they were positively identified with polyclonal antibodies instead (12). Anti-OspA in combination with anti-Flagellin may be an exemplary choice in the analysis of either nucleic acid data or IFA, as these two proteins constitute one-third of the total protein content of a spirochete during early Lyme disease (54, 55). The gene expression profile of long-term persisters within a host is as yet unknown.

Recently, another study was published in which Borrelia spirochetes appeared to be present in the form of biofilms in human brain specimens of a chronic Lyme disease case. This study refers to the usage of a monoclonal antibody that is specific for *B. burgdorferi* sensu stricto (56), yet there was no reference to a commercial source or a research laboratory. The methodology section of the paper cites articles that used a conjugated version of rabbit-polyclonal antibodies which target Borrelia spirochetes. The study neglected to include controls testing cross-reactivity of the antibodies used, so it is difficult to determine the validity of the IFA and to repeat the assay. The authors, however, indicated that *Borrelia* sequences were identified from the tissues through metagenomics sequencing.

In the study reported here, we used primers that target internal transcribed spacer region (ITS) of the bacterial ribosomal RNA. Although the protein coding regions often have a higher specificity compared to ribosomal markers (57), low PCR amplification, integrity of the tissue sample, and low copy number eliminated them as candidates for the PCR assay of our human autopsy specimens. Previously, 16S rRNA gene was utilized for rapid detection and identification of Borrelia species considering its ubiquity among all the members of the Borrelial genus and almost all bacteria (58). However, this 16S rRNA gene would be very difficult to differentiate between species of Borrelia because of its high sequence similarity. To differentiate *Borrelia burgdorferi* from other species, we utilized nested PCR. According to a BLAST search, these primers matched 100% with different isolates of *B. burgdorferi* and didn't align with any other bacteria or host species except, the *Borrelia* species *finlandensis*. According to a recent study in which 7,292 clinical specimens were tested for Borrelia species in US patients, five different species of Borrelia were identified and the species *finlandensis* was not one of them (59). Most recently, a group that analyzed the microbiomes of ticks collected from the states of New York and Connecticut identified only two Borrelia species, *B. burgdorferi* and *B. miyamotoi*, in adult *Ixodes scapularis* ticks (60). Out of 197 ticks that were analyzed, *B. burgdorferi* was detected in 111 (56.3%) of the individual ticks and *B. miyamotoi* in 10 (5.07%) ticks. Among these 10 ticks, seven ticks harbored both species (60). Considering the geographical location and the environment of the Lyme case used in this study and the tick microbiome study, designing primers that are sensitive and specifically detect *B. burgdorferi* was of utmost importance.

Given the disparity in findings over multiple studies, having multiple methodologies to evaluate specimens for Bb should significantly strengthen any results. Studies suggesting a role for Bb in dementia have been published previously by (38, 46, 47, 61, 62), but negative findings for Borrelia spirochetes have also been reported by others as mentioned above (43, 63). Our studies here represent a major improvement in methodology– both in terms of microbial probing techniques and in numbers of brain samples.

In this report, we provide methodology which succeeded in identifying persistent Borrelia in the CNS of a deceased woman with a history of Lyme disease. This patient did not meet full diagnostic criteria for neuroborreliosis, as it was never demonstrated that she had *B. burgdorferi*- specific intrathecal antibody production, nor did her CSF show lymphocytosis. While she did have 4 IgG Bb-specific IgG bands in her CSF when assessed by Western blot, specific intrathecal production which requires a comparison of serum and CSF by a diagnostic ELISA was never assessed. The lack of CSF lymphocytosis may reflect the prior extensive antibiotic therapy. Our molecular results however confirm *B. burgdorferi* invasion of the central nervous system. An earlier lumbar puncture at the time of the initial cognitive decline and prior to the intravenous antibiotic therapy may have confirmed the diagnosis of neuroborreliosis; this case highlights the clinical importance of CSF studies before initiating antibiotic therapy for presumed neurologic Lyme disease. Her initial good response to the IV ceftriaxone suggests a microbial infection was being treated, or that inflammation was dampened. The decline thereafter suggests either that persister Borrelia were present that are now known not to remit with standard antibiotic therapy (6, 12), that an irreversible neurodegenerative process had been triggered by the prior *B. burgdorferi* infection, or that an unrelated neurodegenerative disorder was present at the same time as the presumed *B. burgdorferi* CNS infection.

A prior case series of patients who developed chronic neurologic Lyme disease in the United States (64) noted that encephalopathy may emerge months to many years after treated erythema migrans and that about 22% of these patients with late neurologic manifestations show an initial improvement in cognition after intravenous ceftriaxone therapy that is followed months later by relapse. Our patient demonstrated severe headache at the time of the EM rash which suggests meningeal inflammation, a symptom profile also reported by 41% of the patients at initial infection in the case series of patients who later developed chronic neurologic Lyme disease. Notably, our patient did have a good response to the antibiotic treatment only to develop a sleep disorder 2 years later and a cognitive disorder 4 years later.

This patient's neurodegenerative disorder demonstrated clinical (REM behavior disorder, visuospatial, and attention problems) and neuropathologic features of a Lewy Body Dementia. The case report raises the question of whether *B. burgdorferi* may play a role in the development of Lewy body dementia. Future studies will be directed at testing more affected subjects and more control subjects in order to substantiate or refute this possible link.

## Data Availability Statement

The original contributions presented in the study are included in the article/Supplementary Material, further inquiries can be directed to the corresponding author/s.

## Ethics Statement

The animal study was reviewed and approved by Tulane University IACUC.

## Author Contributions

SG developed methodology, performed all Borrelia detection techniques, and contributed significantly to writing the report. GR provided clinical assessment of the patient. AD performed pathology and consultation. BF provided clinical data, procurement of samples, and consultation. ME provided resources, consultation, and manuscript preparation. All authors contributed to the article and approved the submitted version.

## Conflict of Interest

The authors declare that the research was conducted in the absence of any commercial or financial relationships that could be construed as a potential conflict of interest.
