# Supplementary material for: Detecting Borrelia Spirochetes: A Case Study With Validation Among Autopsy Specimens
Source: Front Neurol. 2021 May 10;12:628045. doi: 10.3389/fneur.2021.628045 (PMC8141553; doi:10.3389/fneur.2021.628045)
Supplement: Supplementary file 1 [file Presentation_1.pptx]

## Slide 1
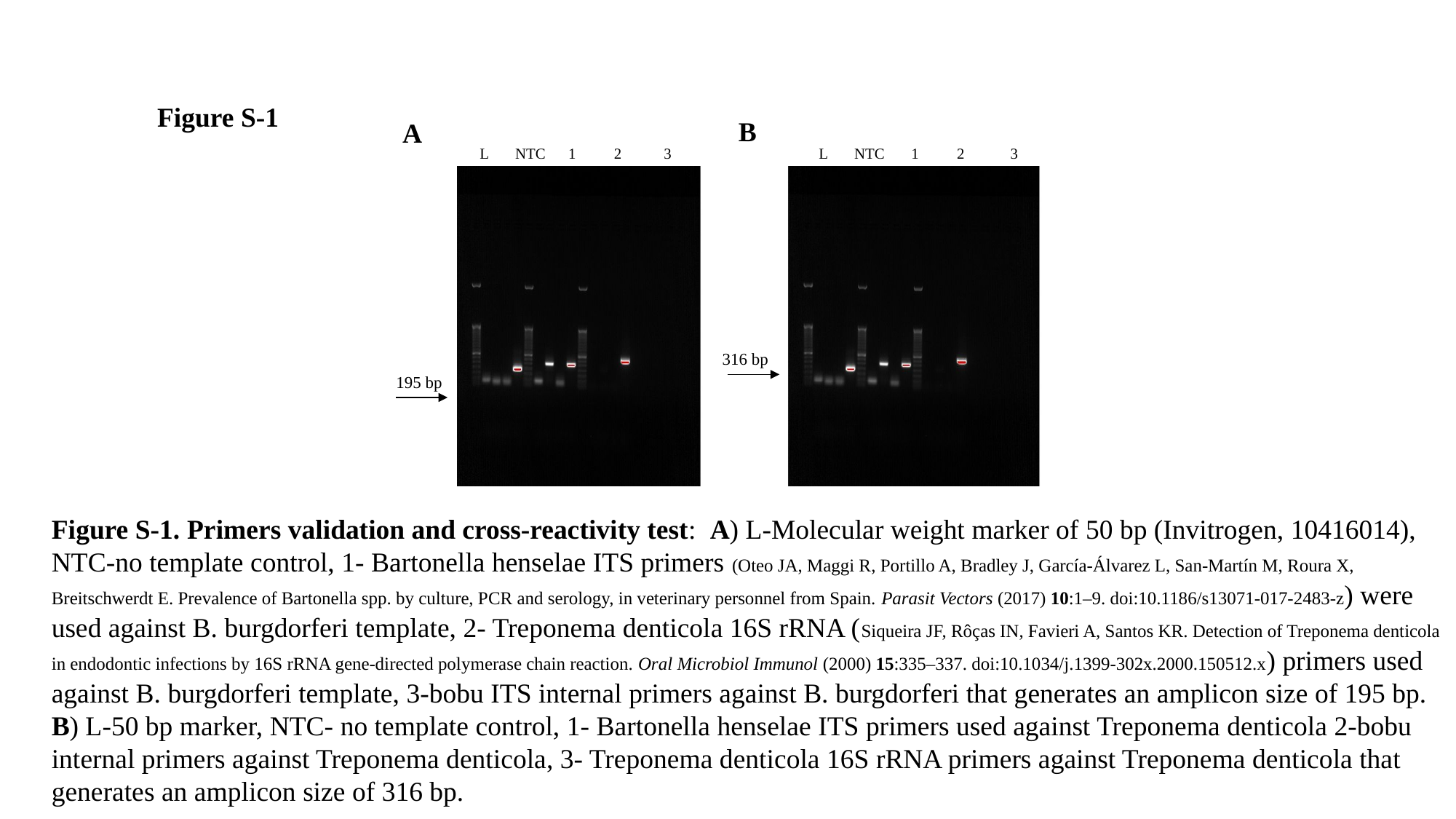

Figure S-1
B
 L NTC 1 2 3
316 bp
A
 L NTC 1 2 3
195 bp
Figure S-1. Primers validation and cross-reactivity test: A) L-Molecular weight marker of 50 bp (Invitrogen, 10416014), NTC-no template control, 1- Bartonella henselae ITS primers (Oteo JA, Maggi R, Portillo A, Bradley J, García-Álvarez L, San-Martín M, Roura X, Breitschwerdt E. Prevalence of Bartonella spp. by culture, PCR and serology, in veterinary personnel from Spain. Parasit Vectors (2017) 10:1–9. doi:10.1186/s13071-017-2483-z) were used against B. burgdorferi template, 2- Treponema denticola 16S rRNA (Siqueira JF, Rôças IN, Favieri A, Santos KR. Detection of Treponema denticola in endodontic infections by 16S rRNA gene-directed polymerase chain reaction. Oral Microbiol Immunol (2000) 15:335–337. doi:10.1034/j.1399-302x.2000.150512.x) primers used against B. burgdorferi template, 3-bobu ITS internal primers against B. burgdorferi that generates an amplicon size of 195 bp. B) L-50 bp marker, NTC- no template control, 1- Bartonella henselae ITS primers used against Treponema denticola 2-bobu internal primers against Treponema denticola, 3- Treponema denticola 16S rRNA primers against Treponema denticola that generates an amplicon size of 316 bp.

## Slide 2
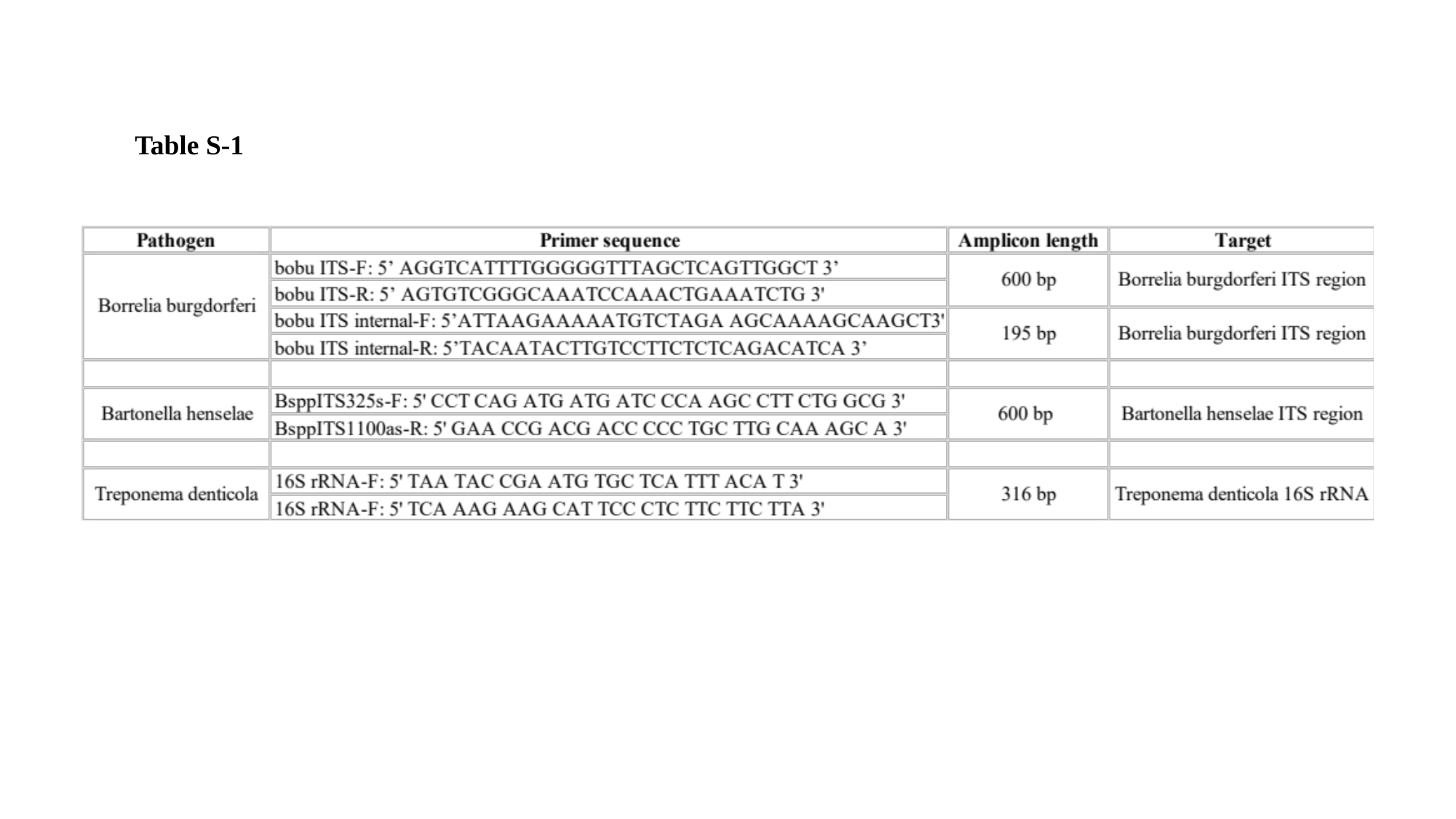

Table S-1

## Slide 3
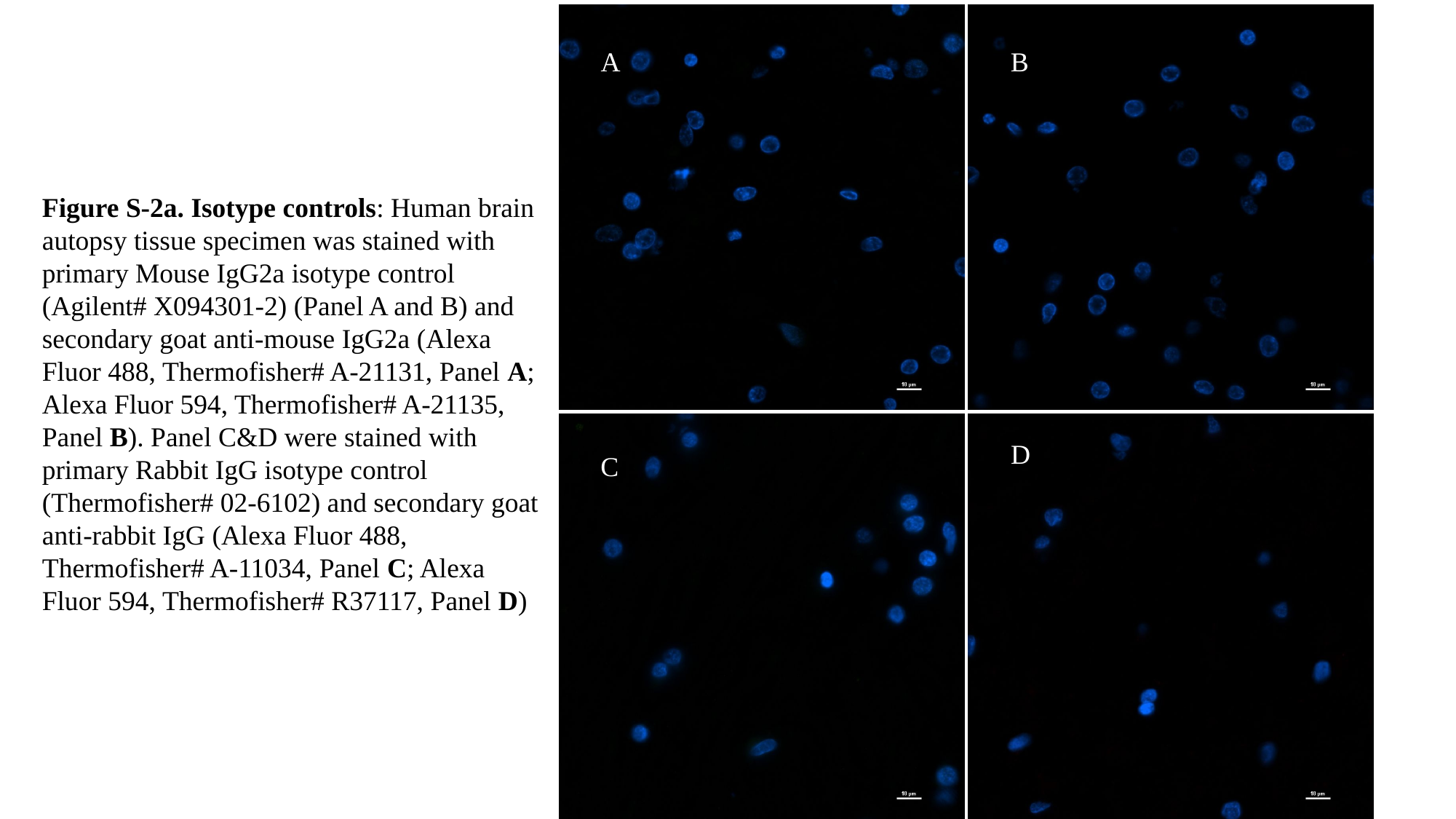

A
B
D
C
Figure S-2a. Isotype controls: Human brain autopsy tissue specimen was stained with primary Mouse IgG2a isotype control (Agilent# X094301-2) (Panel A and B) and secondary goat anti-mouse IgG2a (Alexa Fluor 488, Thermofisher# A-21131, Panel A; Alexa Fluor 594, Thermofisher# A-21135, Panel B). Panel C&D were stained with primary Rabbit IgG isotype control (Thermofisher# 02-6102) and secondary goat anti-rabbit IgG (Alexa Fluor 488, Thermofisher# A-11034, Panel C; Alexa Fluor 594, Thermofisher# R37117, Panel D)

## Slide 4
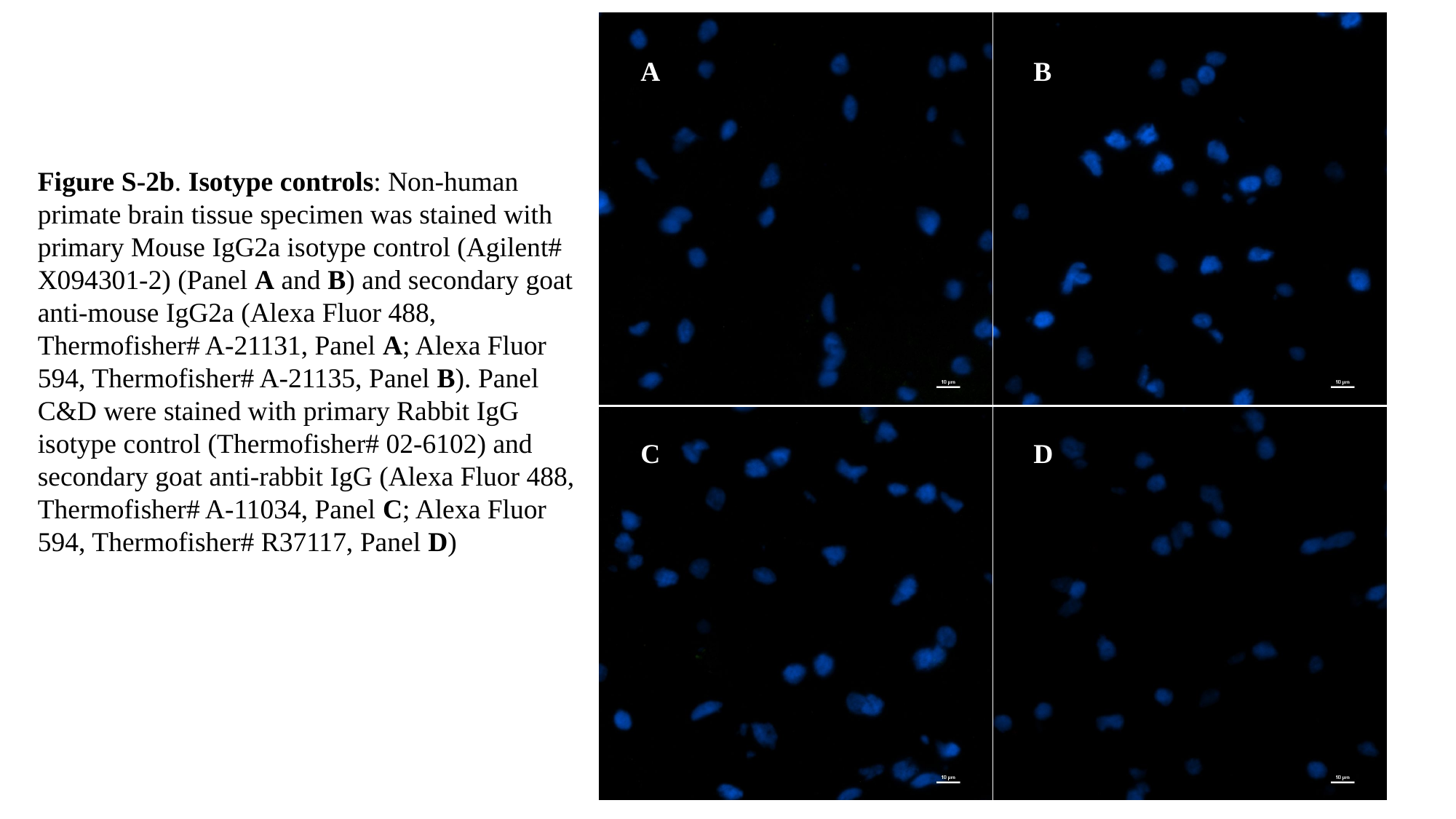

A
B
Figure S-2b. Isotype controls: Non-human primate brain tissue specimen was stained with primary Mouse IgG2a isotype control (Agilent# X094301-2) (Panel A and B) and secondary goat anti-mouse IgG2a (Alexa Fluor 488, Thermofisher# A-21131, Panel A; Alexa Fluor 594, Thermofisher# A-21135, Panel B). Panel C&D were stained with primary Rabbit IgG isotype control (Thermofisher# 02-6102) and secondary goat anti-rabbit IgG (Alexa Fluor 488, Thermofisher# A-11034, Panel C; Alexa Fluor 594, Thermofisher# R37117, Panel D)
D
C

## Slide 5
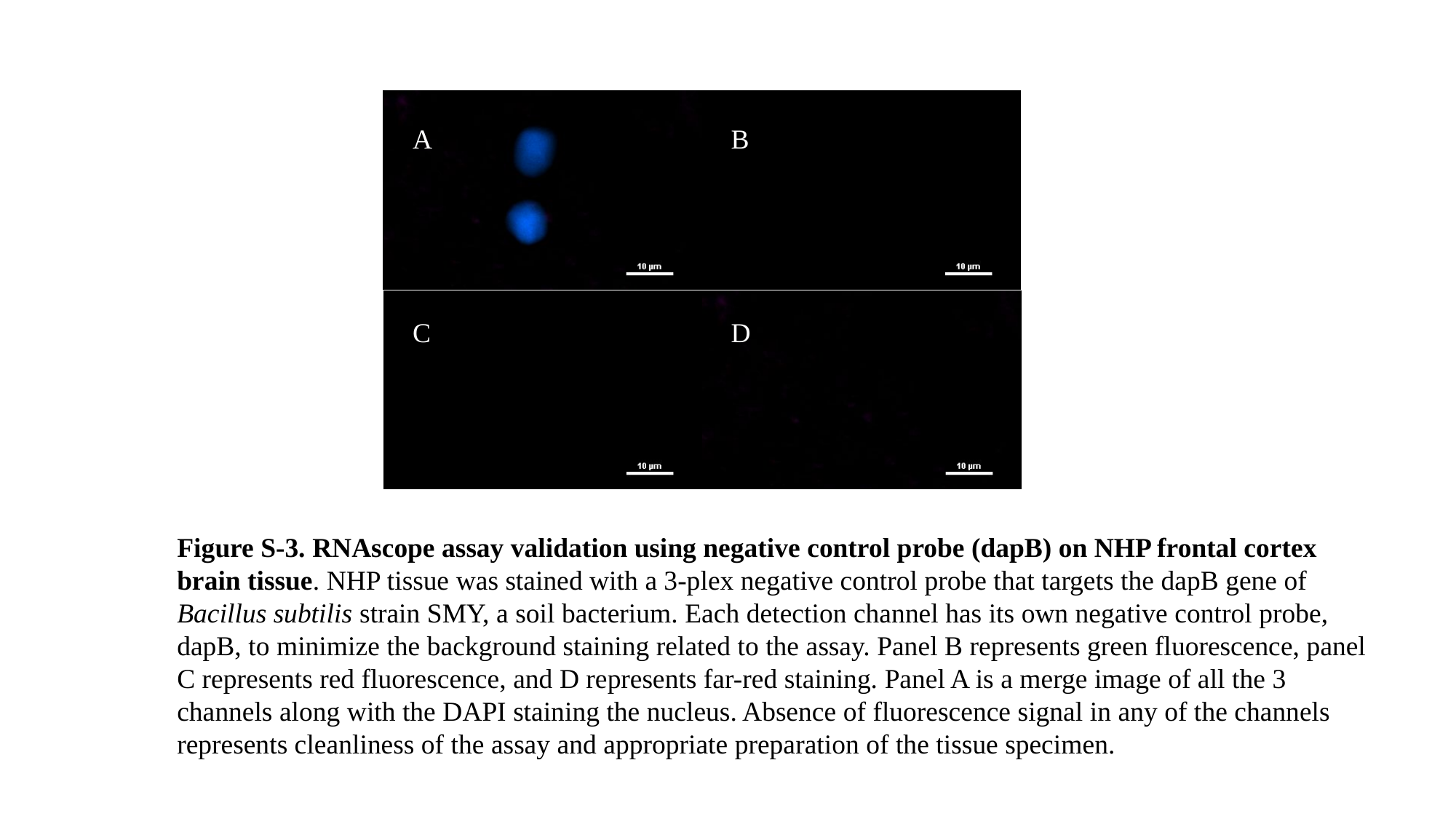

A
B
C
D
Figure S-3. RNAscope assay validation using negative control probe (dapB) on NHP frontal cortex brain tissue. NHP tissue was stained with a 3-plex negative control probe that targets the dapB gene of Bacillus subtilis strain SMY, a soil bacterium. Each detection channel has its own negative control probe, dapB, to minimize the background staining related to the assay. Panel B represents green fluorescence, panel C represents red fluorescence, and D represents far-red staining. Panel A is a merge image of all the 3 channels along with the DAPI staining the nucleus. Absence of fluorescence signal in any of the channels represents cleanliness of the assay and appropriate preparation of the tissue specimen.

## Slide 6
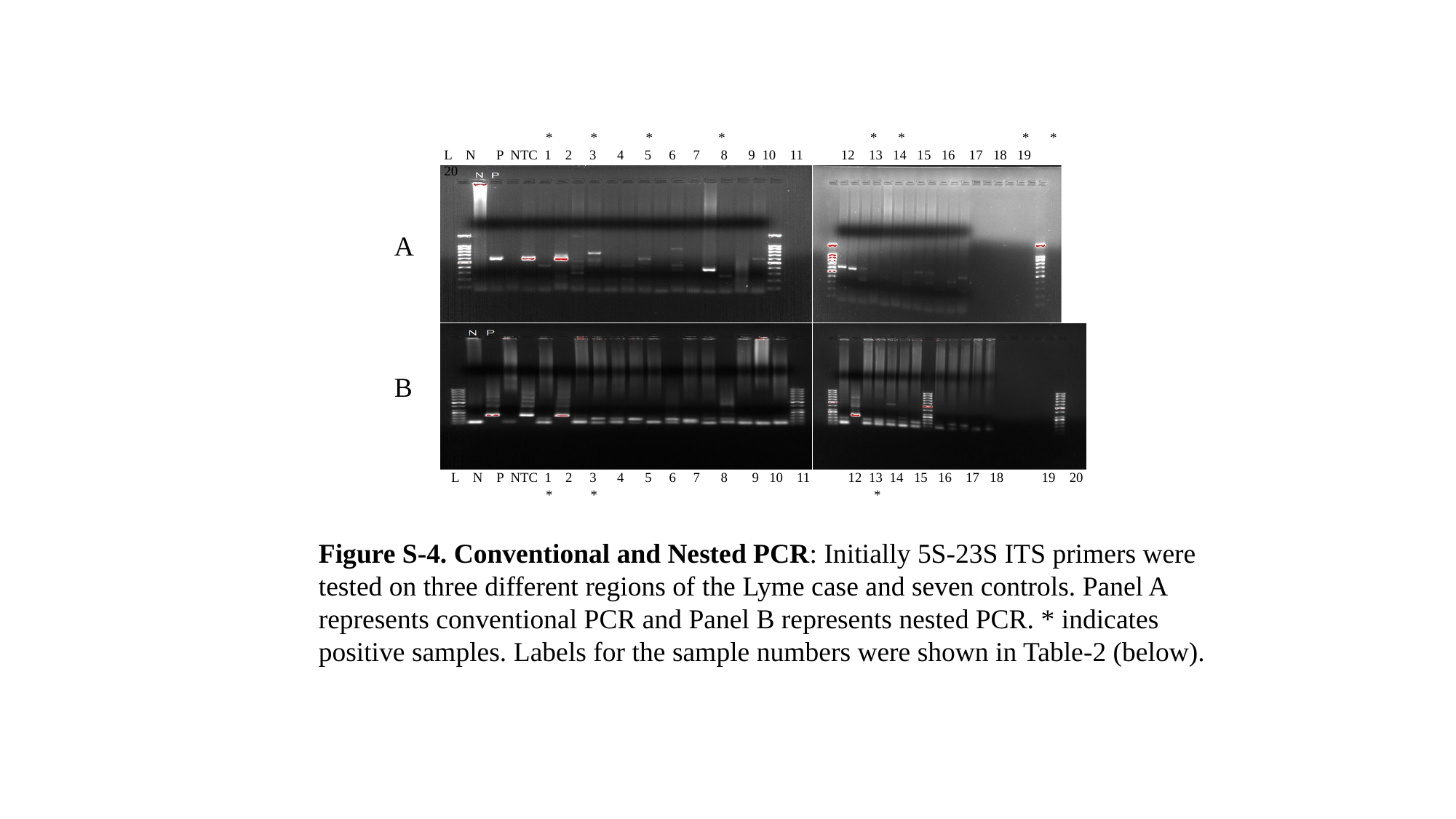

* * * * * * * *
L N P NTC 1 2 3 4 5 6 7 8 9 10 11 12 13 14 15 16 17 18 19 20
L N P NTC 1 2 3 4 5 6 7 8 9 10 11 12 13 14 15 16 17 18 19 20
* * *
A
B
Figure S-4. Conventional and Nested PCR: Initially 5S-23S ITS primers were tested on three different regions of the Lyme case and seven controls. Panel A represents conventional PCR and Panel B represents nested PCR. * indicates positive samples. Labels for the sample numbers were shown in Table-2 (below).

## Slide 7
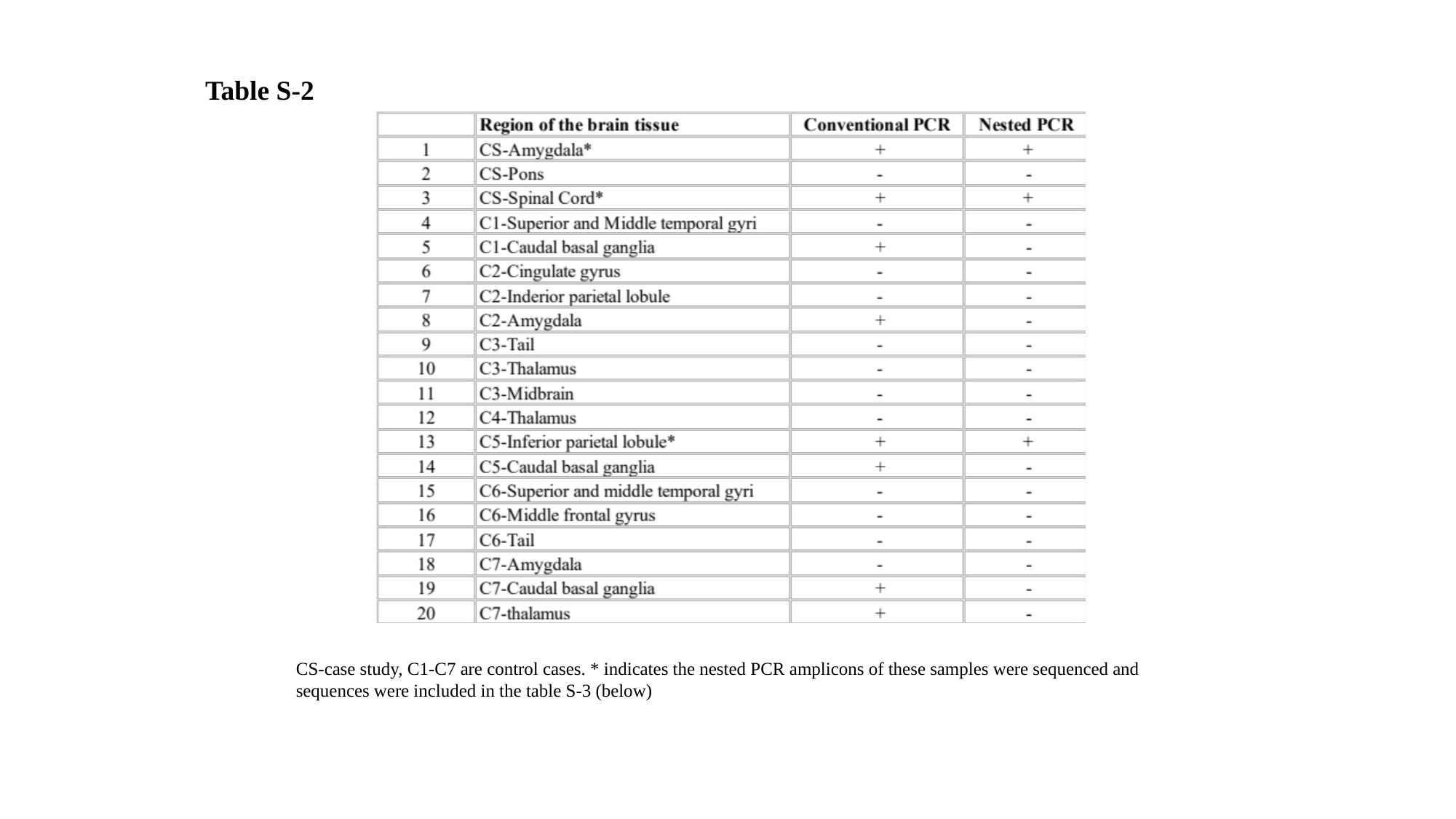

Table S-2
CS-case study, C1-C7 are control cases. * indicates the nested PCR amplicons of these samples were sequenced and sequences were included in the table S-3 (below)

## Slide 8
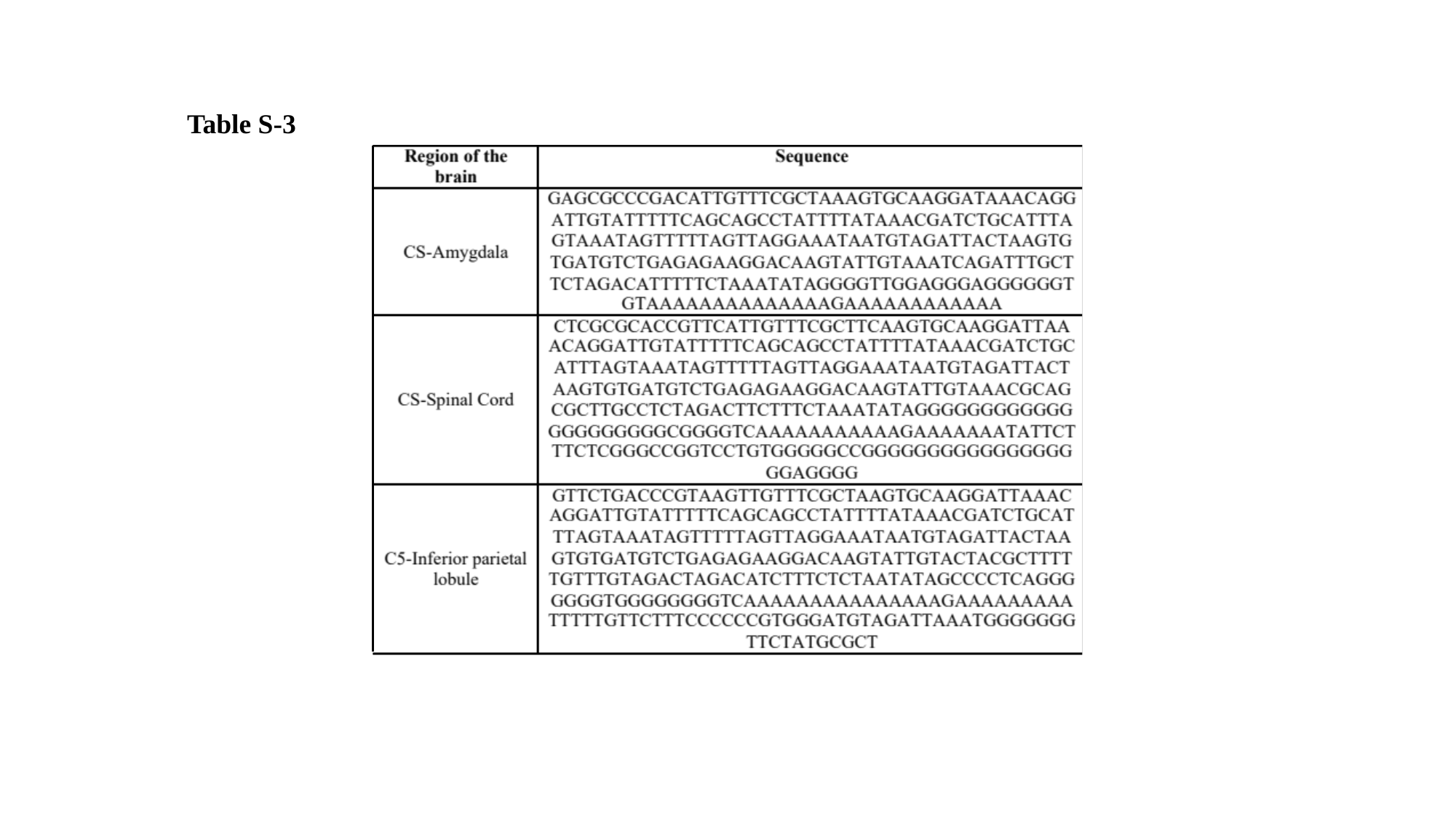

Table S-3
